# Effect of subclinical depression on moral judgment dilemmas: a process dissociation approach

**DOI:** 10.1038/s41598-022-24473-2

**Published:** 2022-11-21

**Authors:** Xiyang Yin, Zijing Hong, Yinjia Zheng, Yali Ni

**Affiliations:** 1 Guangzhou Huashang Vocational College, Center of Mental Health Education and Counseling, Guangzhou, 511300 People’s Republic of China; 2 Jinan University, School of Management, Guangzhou, 510632 People’s Republic of China; 3 Department of Psychology, The Fifth Affiliated Hospital of Guangzhou Medical University, Guangzhou, 510170 People’s Republic of China

**Keywords:** Human behaviour, Depression

## Abstract

Dual-process theory of moral judgment indicates that moral decision-making is guided by emotional or cognitive processing, competing with each other. While emotional processing overwhelms cognitive processing, individuals preferentially make deontological judgments. Further, while cognitive processing dominates emotional processing, individuals preferentially make utilitarian judgments. This theory predicts that individuals with subclinical depression associated with emotion regulation deficits may deliver more utilitarian judgments. Experiment 1 indicated that higher depressive symptoms predicted utilitarian judgment. However, previous studies have not determined why individuals with subclinical depression make a utilitarian judgment. Thus, Experiment 2 employed the process-dissociation approach, which can separately measure the relative strength of individual deontological and utilitarian inclinations. Deontological inclination (parameter *D*) was associated with emotional processing, whereas utilitarian inclination (parameter *U*) was related to cognitive processing. In Experiment 2, the two groups (higher depressive symptoms/minimal depressive symptoms) completed the moral task of the process-dissociation approach to investigate the underlying mechanism. There was a significant interaction effect between the group and parameter of process dissociation. Parameter *D* in the higher depressive symptoms group was weaker than in the minimal depressive symptoms group. Individuals with subclinical depression bias their utilitarian judgments by making fewer deontological moral judgments.

## Introduction

“*I know only that what is moral is what you feel good after and what is immoral is what you feel bad after*.”

Ernest Hemingway

For millennia, philosophers have debated whether emotions contribute to recognizing, interpreting, and evaluating behavior as moral or immoral^1,2^. In recent years, philosophers have held a view that is consistent with the dominant opinion of emotion in psychologists, which argues that emotions play an essential role in human development and growth of moral character^1^.

Social psychologists have long been concerned about the decision of sacrificial minorities in moral dilemma^3^. The dual-process theory of moral judgment indicates that moral decision-making is guided by emotional or cognitive processing in moral dilemmas, competing with each other^3^.If emotional processing overcomes cognitive processing when individuals face moral dilemmas, they tend to make a deontological judgment (e.g., hurting others is inappropriate, regardless of the behavioral outcomes). In instances where cognitive processing overcomes emotional processing when individuals face moral dilemmas, they tend to make a utilitarian judgment (e.g., hurting others is appropriate if the behavior enhances the well-being of the greatest number of people). This is a measurement of utilitarianism and deontology in the moral dilemma paradigm. For instance, in the trolley dilemma, an uncontrollable trolley is about to collide with five men on the track, whereas there is only one person on the other track. When facing this moral dilemma, you must decide to make a utilitarian judgment by pushing a lever to switch the trolley to the other track, killing one person as opposed to doing nothing and allowing the five men to die, thus making a deontological judgment^4,5^. During the process of moral reasoning, individual differences in ethical principles may lead to the adoption of disparate moral judgments. On the one hand, based on cognitive moral reasoning, individuals with utilitarian principles in a sacrificial moral dilemma tend to take moral actions where the well-being of the greatest number of people is given priority. On the other hand, individuals with deontological principles disagreed regarding sacrificing an innocent person for the greater good (killing one person to save the other five men is morally wrong), emphasizing the role of emotion in evaluating actions^5,6^.

According to Greene’s theory, the dual-process theory postulates that emotional response intuitively arises from the aversive emotion of an individual’s sacrifice in such dilemmas, perceiving the consequences of such moral actions and judging their moral acceptance^7^. In agreement with the idea that emotions contribute to moral judgment, Haidt proposed a social intuition model (SIM model) that highlighted the role of emotion in the decision-making process, suggesting that moral decision-making relies heavily on automatic emotional intuition^8^. Emotion responses to actions of actual harm that may lead to deliberate harmful behavior resulting in distress are immoral^9,10^. There is a connection between affective processes and responses to moral dilemmas in clinical or subclinical populations. Compared to healthy controls, clinical evidence from a patient with an injured ventromedial prefrontal cortex showed that the injury resulted in impaired emotional intuition. This evidence supports that a bias to utilitarian consideration contributes to moral judgment as the brain’s emotional system is impaired^11^. Similar findings have been reported in subclinical populations with emotional deficits that predict utilitarian judgement^12^, such as individuals with high levels of alexithymia^13^.

Evidence from clinical patients has shown that individuals who are clinically depressed have impaired processing of moral emotions compared to healthy individuals^14^. Their neuroimaging study showed that depressed patients with bipolar disorder (BD) significantly differed from healthy controls in their neural network when processing in a go-no go task with morally-valence moral decision^14^. One recent study, using the moral dilemma task, also found a more utilitarian judgment in euthymic bipolar disorder patients than in healthy controls^15^. However, a divergent result found differences between only manic bipolar disorder patients (not euthymic) and healthy controls in utilitarian judgment^16^. This might be because the sample size (including only 26 participants) was too small to limit statistical power^15^. Generally, atypical moral judgments are associated with emotional state dependence. Most studies have focused on the association between utilitarian judgment and clinical BD, but few studies have focused on individuals with depression in the public population. Subclinical depressive symptoms were that the individuals refer to a state with certain depressive symptoms, but does not meet all the diagnostic criteria for a major depressive disorder^17^. Furthermore, subclinical depressive symptoms were more prevalent among college students^18^. Thus, there is a need for further studies with more comprehensive samples to explore the relationship between individuals with high depressive symptoms and utilitarian judgment among college students. Additionally, previous evidence has shown that 10–20% of people in the general population have alexithymia traits that frequently co-occur with depression, 26–50% of which overlap with depressive disorders^19,20^. Alexithymia is mainly reflected in defects in emotional processing, and those who are diagnosed with alexithymia cannot recognize and verbalize emotional feelings^21^. Given the close relationship between alexithymia and depressive symptoms^20^, alexithymia may be a confounding factor in the association between depressive symptoms and utilitarian judgment. However, previous researches have merely considered utilitarian judgment in depressive symptoms^14,15^ or alexithymia trait separately^22,23^. There is a need to investigate how alexithymia trait and depressive symptoms contribute to utilitarian judgment. The confounding factors of alexithymia traits should be further investigated to clarify the association between depressive symptoms and utilitarian judgment. Therefore Experiment 1 investigated the separate contributions of alexithymia traits and depressive symptoms to utilitarian judgment in the general population.

The dual-process model suggests that emotional processing and deliberative reasoning represent two separate psychological systems that drive deontological and utilitarian tendencies^4,5^. Previous studies have not determined whether individuals with depression made biased utilitarian judgments based on psychological processing or dual systems. Researchers believe that the key to solving this problem is finding a method to effectively measure emotional and cognitive processing in moral judgment^4,24^. This paradigm was first used to separate the roles of implicit and explicit processing in the memory process and then applied to the investigation of the psychological mechanism of uncertain decision-making and moral judgment, which implies that the dual-process model also influences in decision-making behavior^4^. In the moral judgment task, researchers assumed the dual psychological systems underlying two moral judgments: outcome of action (parameter *U*) and social norm (parameter *D*), respectively, regarding the utilitarian and deontological inclination^25^. The process-dissociation paradigm (PDA) was employed to assess the relative strength of participant deontological and utilitarian judgment in incongruent and congruent dilemmas independently. To satisfy the requirements of the paradigm of process separation, the incongruent dilemmas in the experimental materials point to the moral dilemma story with a strong emotional conflict. In contrast, the congruent dilemmas point to the non-dilemma moral level without apparent emotional strife. This may weaken the emotional experience in congruent dilemmas, leading the participants to make utilitarian (driven by cognitive processing) judgments in different situations. In incongruent dilemmas, individuals applying utilitarian principles indicated harmful actions, whereas individuals applying deontological principles rejected destructive behavior. In congruent dilemmas, individuals based on deontological or utilitarian tenets are compatible with considering harmful behaviors. Based on the above analysis, this study screened moral dilemma stories that could effectively trigger psychological conflict, derived from the studies of Hayakawa et al. as experimental materials^24^. Based on the above analysis, Experiment 2 explored whether individuals with depression biased their utilitarian judgment based on psychological processing systems or dual systems via the process-dissociation paradigm. Moreover, participants were recruited in the higher depressive symptoms and minimal depressive symptoms groups. The dual-process model postulated that deficits in emotional processing could weaken deontological judgment. Thus, it was hypothesized that the higher depressive symptoms group would deliver fewer deontological judgments and more moral acceptance than the minimal depressive symptoms group.

## Experiment 1

### Methods

Participants. 1849 participants were recruited in Experiment 1 from aged 18 to 20, and 1775 participants (822 female) voluntarily logged onto the Questionnaire Star platform (http://www.wjx.cn/) and completed the online survey. Completion of questionnaire was taken as informed consent. Each participant was informed that personal data was kept anonymous and only be used for research purposes. Before submission, it was required to answer all the questions. The participants had the freedom to quit the survey at any time. Ten min were required to finish this survey. The current research involving human research participants have been performed in accordance with the Declaration of Helsinki. Furthermore, the current research was approved by the research ethics committee of Guangzhou Huashang Vocational College, in accordance with the regulations of the World Medical Association. The college students took part in the survey during the mental health education sessions between November 1, 2020, and December 30, 2020. Completing the survey, each student can get a pen costed $0.15.

### Measures and procedure

Depressive level. The Chinese version of the Patient Health Questionnaire (PHQ-9) measures depression^26^. PHQ-9 total score ranges from 0 to 27 for calculating all the nine items to assess the severity of a depressive episode in the general population including 9 items with a 4-point Likert scale from 0 (not at all) to 3 (nearly every day). The PHQ-9 has good psychometric properties with internal consistency.

Alexithymia. The Chinese version of the 20-item Toronto Alexithymia Scale (TAS-20) was used to measure the alexithymia trait, including 20 items with a 5-point Likert scale from 1 (strongly disagree) to 5 (strongly agree), which scores > 60 suggesting a high degree of alexithymia, and scores < 52 suggesting a definite absence of alexithymia^27^. It was reliable and valid for the sample of Chinese undergraduates.

Utilitarian judgment. An updated set of original versions of dilemmas^4,24^, such as the footbridge dilemmas and fumes dilemmas, were used in the experiment (see the example in Table 1. Further information is provided in the supplementary information). The Chinese version was adapted according to Chinese culture, which selected seven moral dilemmas (see Table 1 for examples). Their psychometric properties were adequate for college students. All dilemmas were framed in first-person form. Participants were instructed to indicate whether the described action was acceptable or not (acceptable/unacceptable) in each of the moral dilemmas, where “acceptable” would be registered as a “utilitarian judgment.” Further information is provided in the supplementary data.

**Table 1:** The moral dilemma task with representative examples.

Moral dilemma task	Text description	Behavioral question
Footbridge dilemmas	An uncontrol tram was running along the track to five workers working on the track. If the tram was allowed to continue, the five workers would be killed. You are standing on the footbridge above the railway track. The footbridge is between the moving tram and the five workers. As it happens, there is a huge stranger next to you. The only way to save the lives of the five workers is to push the stranger down from the overpass and let him fall into the middle of the track, so as to stop the progress of the tram. If you do (nature of action), the stranger will die, but the five workers will be saved (outcome of the proposed action)	Would you (nature of action) in order to (outcome of the proposed action)? A. acceptable B. unacceptable
Fumes dilemmas	You are the night duty watchman of the hospital. As a result of an accident, deadly smoke entered the hospital's ventilation system. There are three patients in one room. In another room there is a single patient. If you do nothing, the smoke will spread to the room containing the three patients and cause their death. The only way to avoid their death was to press the control switch (nature of action), which makes the smoke bypass the rooms containing the three patients. If you done, smoke will enter the containing one single patient, causing his death, the three patients will be saved (outcome of the proposed action)	

The study designs and experimental procedures in Experiments 1 and 2 were all approved by the Research Ethics Board of Guangzhou Huashang Vocational College.

### Data analysis

Statistical analysis was also carried out using SPSS 25.0. Univariate descriptive analyses were performed, as well as correlation analyses among all the variables. To further examine the contribution of depressive symptoms and alexithymia in utilitarian judgment, we used the multiple regressions (Stepwise regression) with depressive symptoms and alexithymia rating as the independent variables and with utilitarian judgment as to the dependent variable.

### Results

The common variance was controlled through Harman’s single-factor test (18.85%), demonstrating that a single factor did not account for the majority of the covariance. These scales reported a satisfied internal reliability (ɑ > 0.6). The depressive level, alexithymia, and utilitarian judgment correlated with each other (see Table 2).

**Table 2:** Overview on the descriptive statistic results of sample (*N* = 1775).

Parameter	Cronbach's α	*M* ± *SD*	Alexithymia trait	Depressive level	Utilitarian judgment
Alexithymia trait	0.88	52 ± 11.33	1	–	–
Depressive level	0.89	13.85 ± 4.42	0.53***	1	–
Utilitarian judgment	0.64	0.30 ± 0.24	0.08***	0.11***	1

*p < 0.05, ***p < 0.001.

A multiple regression analysis was conducted to assess if TAS-20 scores contributed to the relationship among depressive symptoms, alexithymia trait, and utilitarian judgment. Regression analyses revealed that only depressive symptoms (PHQ-9: *M* ± *SD* = 13.85 ± 4.42) can predict the utilitarian judgment but not the alexithymia trait (TAS-20 scores: *M* ± *SD* = 52 ± 11.33), *F* (2, 1772) = 5.53, *SE* = 0.03, *t* = 7.32, *p* < 0.05. Firstly, high depressive symptoms in individuals were associated with an increase in utilitarian judgment, *t* = 3.05, *p* < 0.05. Secondly, no association was reported between alexithymia and utilitarian judgment, *t* = 0.30, *p* > 0.05. Consequently, higher depressive symptoms predict utilitarian judgment in the general population.

## Experiment 2

Experiment 1 concluded that depressive symptoms, but not alexithymia, affect utilitarian judgment; however, previous studies did not explain the underlying mechanism between depression and moral judgment. Hence, we used a mixed 2 × 2 design, with one within-subjects factor (moral judgment: deontological, utilitarian) and one between-subjects factor (groups: higher depressive symptoms group/minimal depressive symptoms group). The process-dissociation paradigm (PDA) was applied to assess the relative strength of participants’ deontological and utilitarian judgment in congruent and incongruent dilemmas separately (the full text of PDA can be viewed in the supplementary information).

In incongruent dilemmas, individuals holding utilitarian principles indicated harmful actions, whereas individuals holding deontological principles rejected destructive behavior. For example, in the dilemmas of car accidents, individuals with utilitarian inclinations tend to harm an older woman to rescue a young mother and kid by shifting the steering wheel. Conversely, individuals with deontological inclinations were dejected to harm the older woman, even if the young mother and child were at risk. In congruent dilemmas, individuals whose thinking was based on deontological or utilitarian principles are compatible with considering harmful behaviors. For example, in the congruent dilemmas of car accidents, one would have to change the steering wheel to save the young mother and child but kill a group of primary school students. In this dilemma, there was no apparent conflict between emotion and rationality, as turning the steering wheel violates morality and cannot save more lives. Therefore, all the participants refused to turn the steering wheel.

The relative strengths of deontological and utilitarian inclinations for each participant were calculated by comparing congruent and incongruent dilemma choices. Therefore, participants were recruited for the higher depressive symptom and minimal depressive symptom groups.

### Methods

Participants. In Experiment 2, estimated sample sizes were calculated using G*Power 3 v3.1.9.7 (http://www.gpower.hhu.de/), yielding a total sample size of 66 participants (33 per group)^28^. The sensitivity power analysis determined the sample size by providing 0.80 power to deter an effect size of ƒ = 0.25. Based on the data mentioned above and considering the sample sizes of previous research, 70 participants from the college (35 per group) between 18 to 20 years of age were recruited^29^. All the participants were right-handed. Before the experiment, 2,600 students completed the Patient Health Questionnaire-9 (PHQ-9). According to their PHQ-9 scores, 35 participants in the higher depressive symptoms group had major depressive symptoms (PHQ-9 > 14), and 35 participants in the minimal depressive symptoms group showed no or mild depressive symptoms (PHQ-9 < 4). All participants offered their written informed consent in accordance with the Declaration of Helsinki before the experiment. Experiment 2 was approved by the research ethics committee of Guangzhou Huashang Vocational College, in accordance with the regulations of the World Medical Association.

### Materials

Depressive level. All the details are described in Experiment 1. No/mild depression: PHQ-9 < 10; Moderate to severe depression: PHQ-9 ≥ 10 (moderate depression: 10–14, major depression: 15–19, severe depression: 20–27)^26^.

Moral judgment task. We employed a process-dissociation task, which included seven incongruent and seven congruent moral dilemmas. The incongruent dilemmas between cognitive conflicts and emotional ones made conflict judgments of dilemmas in moral decisions, which were selected from Experiment 1. The congruent dilemmas were matched with the incongruent dilemmas, which modified the incongruent dilemmas to minimize moral conflict. Seven dilemmas comprising congruent and incongruent versions were designed to measure participants’ utilitarian and deontological principles. For instance, in a car accident involving a congruent dilemma, participants were instructed to judge whether turning and hurting the schoolchildren to avoid hitting a young mother and her child was acceptable. In this scenario, individuals with utilitarian and deontological principles refused to hurt the children. Correspondingly, the calculation method follows the process-dissociation task requirement^4,24^. Based on the proportion of “unacceptable” responses by the participants, we calculated the proportion of each participant in the seven incongruent and congruent scenarios. The utilitarianism (*U*) and deontology (*D*) parameters are calculated as follows: *U* = *p* (unacceptance/congruent) − *p* (unacceptance/incongruent), *U* ranging from − 1 to 1; *D* = *p* (unacceptance/congruent) or (1 − *U*), *D* ranging from 0 to 1. A high *U* score suggested endorsing harmful behaviors, while a high *D* score suggested a rejection of harmful behaviors.

### Procedure and design

The participants were invited to sit in a quiet chamber in front of a 24-inch monitor. E-Prime 2.0 was used to present the moral stories and response acquisition, and the texts were presented in white Arial font on a gray background (RGB, 80, 80, 80). Before the experiment, the participants were given clear instructions for the task. The text was shown on a single screen for each moral dilemma in random order. Participants could read the text at their own pace without time limits and change the question via the “space” key. Each question was subsequently displayed on a single screen within a text. First, participants were asked to select whether they would perform (“*acceptable*”) via the “F” key or refuse to perform (“*unacceptable*”) via the “J” key on the keyboard, wherein utilitarian judgments led participants to select “*acceptable*.” Participants also rated the question “How morally acceptable is it for you to [nature of action] to [outcome of action] (e.g., how morally acceptable is it for you to kill one person to save the five persons by pulling the switch),” using a 7-points Likert scale (1 = not at all, 7 = very much) via the “1 to 7” number key.

### Results

Descriptive Statistics. The two parameters did not correlate with each other (*r* = 0.02, *p* < 0.05), which indicates that the dual processes of moral judgment are separate. There was a significant difference reported in PHQ-9 scores between the higher depressive symptoms and minimal depressive symptoms groups, *t* (68) = 17.23, *p* < 0.00.

**Figure 1:**
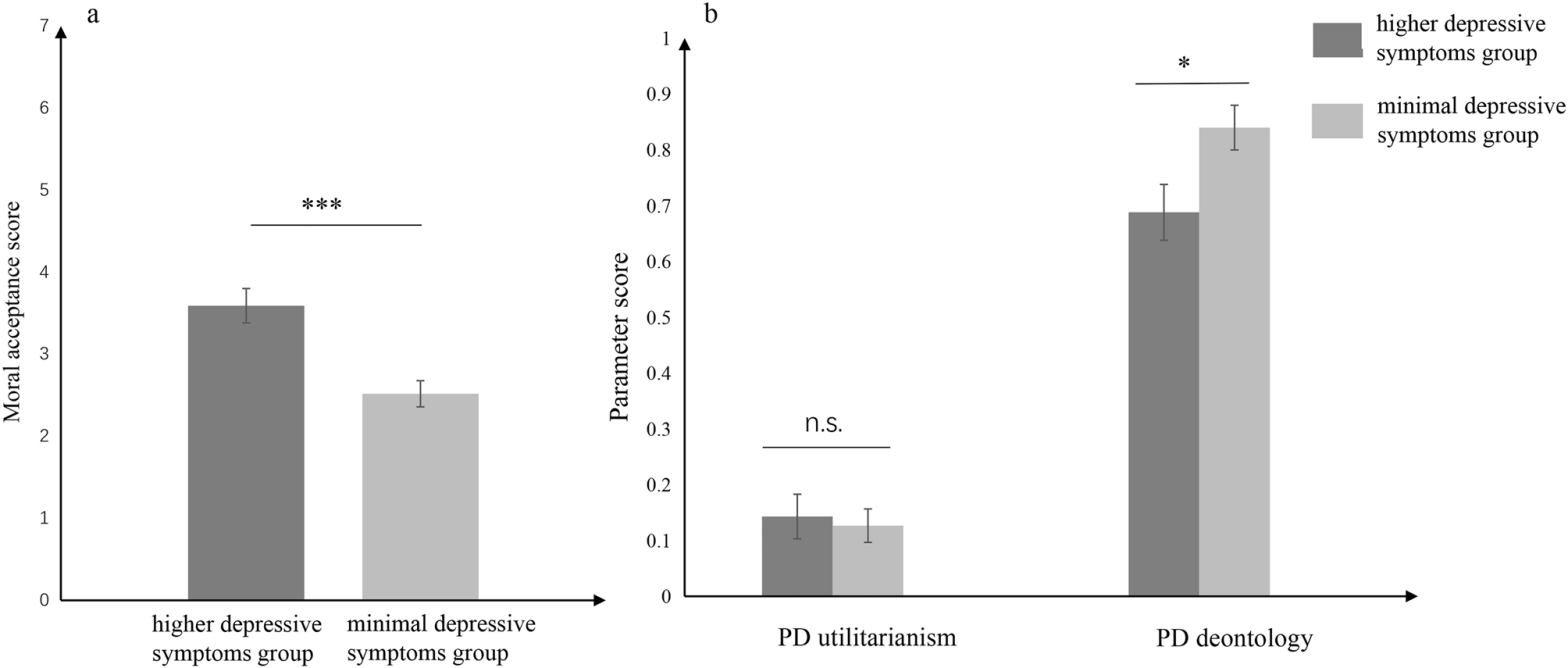
(**a**) The moral acceptance score for the higher depressive symptoms group/minimal depressive symptoms group. (**b**) Mean standardized parameter *U* and parameter *D* for the individuals with depression and healthy controls. Error bars indicated standard errors. *p < 0.05, *** *p* < 0.05, and n.s. indicated no-significant differences as *p* > 0.05.

The Moral Acceptance of incongruent dilemma. In the case of moral acceptance, a significant difference was found using independent t-test (*t* (1, 68) = 4.28, *p* < 0.01, Cohen’s *d* = 1.02) between the higher depressive symptoms and minimal depressive symptoms groups (See below Fig. 1a). The moral acceptance of higher depressive symptoms group (*M* ± *SD* = 3.59 ± 1.22) was significantly higher than the minimal depressive symptoms group *(M* ± *SD* = 2.48 ± 0.94) in the incongruent scenario.

Process-Dissociation Analysis. The parameter *U* and *D* were employed to determine the effect on moral judgment. Experiment 2 designed a mixed 2 × 2 design, with one within-subjects factor (parameter: *U*, *D*) and one between-subjects factor (groups: higher depressive symptoms group/minimal depressive symptoms group). A repeats-measures analysis of variance was conducted on a group with the between-subjects factor and the moral judgment of process-dissociation as a within-subject variable (see below Fig. 1b). There was a significant main effect of the parameter, *F* (1, 68) = 251.63, *p* < 0.00, η^2^ = 0.63. The parameter *D* (*M* ± *SD* = 0.76 ± 0.27) of process-dissociation significantly higher than the parameter *U* (*M* ± *SD* = 0.14 ± 0.27). Although the main effect of groups was not significant (*F* (1, 68) = 2.68, *p* = 0.11, η^2^ = 0.00), but its interaction effect between the group and the parameter of process-dissociation was significant (*F* (1,68) = 4.49, *p* = 0.04, η^2^ = 0.01). In the analysis of the simple effects, deontological judgment (parameter *D*) in the higher depressive symptoms group (*M* ± *SD* = 0.69 ± 0.29) was significantly weaker than the minimal depressive symptoms group (*M* ± *SD* = 0.84 ± 0.24). However, there was no significant difference between the higher depressive symptoms group (*M* ± *SD* = 0.14 ± 0.26) and minimal depressive symptoms group (*M* ± *SD* = 0.13 ± 0.16) in the utilitarian judgment (parameter *U*).

## Discussion

We aimed to determine whether depressive symptoms influenced the effect of the utilitarian judgment. Depressive symptoms were rated as the independent variable, and utilitarian judgment was rated as the dependent variable. These results suggested that depressive symptoms can predict utilitarian judgment rather than alexithymia. Experiment 2 further investigated the internal mechanism by utilizing the process-dissociation approach to separately measure the relative strength of deontological and utilitarian judgment of the individual. The following results indicate that individuals with subclinical depression tend to make fewer deontological judgments and more morally acceptable decisions when faced with moral dilemmas. The present study adds to the existing literature in another manner wherein depressive symptoms with emotion deficits lead to utilitarian judgment in the general population, implying that individuals with depressive symptoms lack emotional reactions when making moral decisions in moral dilemmas.

Experiment 1 aimed to determine whether depressive symptoms and alexithymia mutually contribute to utilitarian judgment. Regression analysis indicated that only depressive symptoms contributed to utilitarian judgment. Are alexithymia and depression distinct or overlapping in moral judgment. Alexithymia is neither necessary nor sufficient in diagnosing depression, nor is it universally present among individuals with depression. Although both alexithymia and depression exhibit emotional dysfunction, it might have masked the differences in alexithymia when participants processed emotional information. Depression has been found to explain the effect of attentional bias toward emotional stimuli but not the same is not true for alexithymia^19^. Crucially, the current findings indicate that alexithymia did not significantly contribute to moral judgment. This stipulates that the depressive symptoms, but not alexithymia, affect utilitarian judgment. In line with our results, researchers found that alexithymia could result in an emotional reaction in the context of moral dilemmas but did not result in moral decision-making^7,13^. Experiment 1 specified that individuals with alexithymia might rely on what they deem socially appropriate when making moral decisions. They could rely upon applying general rules about what is “acceptable” and what is “unacceptable.” Researchers have argued that the inability of persons with alexithymia to express their own emotional experiences might only become salient when such individuals are asked to express their feelings spontaneously^30^. This finding reported a significant distinction between the alexithymia and depressive symptoms with nonclinical samples in this study, which is consistent with previous reports of a distinction between alexithymia and depression^19,21^. Second, consistent with the dual-process model of moral judgment, the results indicated that individuals with depression failed to integrate their emotional responses^31^, which biased their moral decisions. More importantly, this intuitive process relies on contextual emotional information^32^. Declined dependence on emotion in those with major depression is consistent with previous reports of decreased emotional bias during moral processing. This evidence further supports the context of a dual-function model of human moral decision-making, in which both intuitive and deliberative processes interact.

In Experiment 2, a process dissociation task was employed to examine the internal mechanism of making moral judgments among individuals with severe depressive symptoms. First, based on deficits in emotional processing, the higher depressive symptoms group involved fewer emotional reactions when hurting another individual in the sacrificial moral dilemma (nobody ought to be sacrificed)^4^. They had decreased emotional concerns regarding others’ harm and the inference of mental state^13^. These results support the hypothesis that the higher depressive symptoms group would deliver fewer deontological judgments and more moral acceptance than the HC group. Previous studies have not evaluated deontological and utilitarian judgments in depressed people^33^. The relationship between depression and moral judgment has not been fully discussed. Therefore, it could not be determined whether the emotional defects of depression would impact moral judgment by affecting either the processing systems or one of them. To fill this gap, Experiment 2 used the process dissociation technique. The current study confirmed that individuals with severe depression are inclined to make fewer deontological moral decisions. The utilitarian tendency was not significantly different between the two groups. The results showed that conventional deontological judgments were considerably higher in the minimal depressive symptoms group than in the higher depressive symptoms group. The group with higher depressive symptoms agreed to accept harmful behavior in moral dilemmas. These results were consistent with the individual conclusions, which suggested that emotional defects in depression cannot integrate the emotional processing system into moral decision-making^34,35^. From a cognitive neuroscience perspective, this experimental effect might be due to the amygdala’s reaction to emotional signals. Further, the ventromedial prefrontal cortex and other areas implicated moral decision-making are thus important targets for future investigation^11,36^. Possibly, among subclinical depressive individuals, morally acceptable judgments were not to enhance the well-being of the majority but due to the reduced deontological tendency that allows them to hurt others when faced with moral dilemmas. When confronted with cognitive conflicts, individual dependent on a system of cognitive control to help them resolve the current conflict they are experiencing^37^. Major depression presented abnormal conflict processing in cognitive conflict tasks^38^, which can arise from this emotional interference and can compromise the ability to complete tasks requiring cognitive control^39^. Particularly, cognitive control resources can regulate and control other cognitive processes during moral decision-making that may be crucial for making moral judgments^5^. Therefore, the depressive symptoms may disrupt processing cognitive control of moral judgment for the emotional information that biased the moral judgment.

In conclusion, this is the first study to explore the association between depression and utilitarian judgment in Chinese culture. Moreover, the deontological inclinations of individuals with depression can influence their moral decisions. Moral judgment is vital for conducting and sustaining social interactions, suggesting that abnormal moral inclinations might increase the social difficulties experienced by depression. In addition, alexithymia did not bias utilitarian judgment, contributing to mixed findings on utilitarian judgment in alexithymia. Owing to the fact that self-reports were employed in the study to assess moral tendency, a comprehensive method (such as physiological indexes) should be used to measure individuals when making moral judgments. Thus, it is essential to explore the neurocognitive mechanisms^40^ by which individuals with depression reduce their deontological inclinations, which can help us understand moral development in individuals with depression.

## Supplementary Information

Supplementary Information 1.

Supplementary Information 2.

## Data Availability

All data generated or analysed during this study are included in this published article (and its Supplementary materials files).
