# Supplementary material for: Effect of subclinical depression on moral judgment dilemmas: a process dissociation approach
Source: Sci Rep. 2022 Nov 21;12:20065. doi: 10.1038/s41598-022-24473-2 (PMC9681861; doi:10.1038/s41598-022-24473-2)
Supplement: Supplementary file 1 — Supplementary Information 1. [file 41598_2022_24473_MOESM1_ESM.docx]

# Supplementary material for

**“**Effect of Subclinical Depression on Moral Judgment Dilemmas: A Process Dissociation Approach**”**

Xiyang Yin^1,*^, Zijing Hong^2^, Yinjia Zheng^3^, and Yali Ni^1,*^

^1^Guangzhou Huashang Vocational College, Department of student affairs, Guangzhou, 511300, PR China

^2^Jinan University, School of Management, Guangzhou, 510632, PR China
^3^The Fifth Affiliated Hospital of Guangzhou Medical University, Department of psychology, Guangzhou, 510170, PR China

*Correspondence should be addressed to: [yinxiyangpsy@foxmail.com](mailto:yinxiyangpsy@foxmail.com) or HSniyali@163.com

## Text S1: Questionnaires used, their internal reliability and validity analysis

*Depressive level*: The Chinese version of the Patient Health Questionnaire (PHQ-9) measures depression. PHQ-9 total score ranges from 0 to 27 for calculating all the nine items to assess the severity of a depressive episode in the general population including 9 items with a 4-point Likert scale from 0 (not at all) to 3 (nearly every day). The PHQ-9 has good psychometric properties with internal consistency. Depressive level were measured using PHQ-9 in all the experiment.

Alexithymia trait: Interindividual differences in subclinical alexithymia were evaluated using The Chinese version of the 20-item Toronto Alexithymia Scale (TAS-20). TAS-20 was used to measure the alexithymia trait, including 20 items with a 5-point Likert scale from 1 (strongly disagree) to 5 (strongly agree), which scores > 60 suggesting a high degree of alexithymia, and scores < 52 suggesting a definite absence of alexithymia. It was reliable and valid for the sample of Chinese undergraduates. Each item consisted of statements about emotional awareness and participants reported their agreement with these statements using a 5-point-Likert scale (1: strongly disagree, 5: strongly agree). TAS-20 has been argued to be the best current measure overall for assessing alexithymia due to its sound reliability, validity, and broad generalizability.

## Internal reliability and validity analysis:

Cronbach's alphas and KMO value were investigated using SPSS24.0.

| Scale | Items | *M* ± *SD* | Cronbach's α | KMO value | *χ* ^2^ | *p* |
| --- | --- | --- | --- | --- | --- | --- |
| PHQ-9 | 9 | 13.85 ± 4.42 | 0.89 | 0.92 | 6308.50 | 0.00 |
| TAS-20 | 20 | 52 ± 11.33 | 0.88 | 0.94 | 15212.03 | 0.00 |

## Text S2: Textual description of moral dilemmas

Utilitarian judgment. An updated set of original versions dilemmas4,24, such as the footbridge dilemmas and the fumes dilemmas, were used in the experiment. The adaptation of the Chinese version was according to Chinese culture, which elected seven moral dilemmas (see Table 1 for examples). Their psychometric properties were adequate for the college student. All the dilemmas were framed in first-person form. Participants were instructed to indicate whether the described action is acceptable or not (acceptable/ unacceptable) in each of the moral dilemmas, where “acceptable” would be registered as a “utilitarian judgment”.

|  | Items | *M* ± *SD* | Cronbach's α | KMO value | *χ* ^2^ | *p* |
| --- | --- | --- | --- | --- | --- | --- |
| Utilitarian judgment | 7 | 0.30 ± 0.24 | 0.64 | 0.74 | 1459.21 | 0.00 |

| Moral dilemma task | Text description | Behavioral question |
| --- | --- | --- |
| 1.Footbridge dilemmas | An uncontrol tram was running along the track to five workers working on the track. If the tram was allowed to continue, the five workers would be killed. You are standing on the footbridge above the railway track. The footbridge is between the moving tram and the five workers. As it happens, there is a huge stranger next to you. The only way to save the lives of the five workers is to push the stranger down from the overpass and let him fall into the middle of the track, so as to stop the progress of the tram.  If you do, the stranger will die, but the five workers will be saved. | Would you (nature of action) in order to (outcome of the  proposed action)?  A. acceptable  B. unacceptable |
| 2.Fumes dilemmas | You are the night duty watchman of the hospital. As a result of an accident, deadly smoke entered the hospital's ventilation system. There are three patients in one room. In another room there is a single patient. If you do nothing, the smoke will spread to the room containing the three patients and cause their death. The only way to avoid their death was to press the control switch, which makes the smoke bypass the rooms containing the three patients.  If you done, smoke will enter the containing one single patient, causing his death, the three patients will be saved. |  |
| 3.Car accident dilemmas | You are driving on a busy city street when a young mother with a child falls in front of your car. At this time, it is too late to brake. Your only hope to avoid an accident is to change the driving direction. Unfortunately, the only place you can turn is a small old lady.  If you change your driving direction to avoid young mother and children, you will seriously crash and even kill the old lady. |  |
| 4.Animal Research dilemmas | You are a product developer in a pharmaceutical company. In order to ensure that drugs are suitable for human use, they should first be tried on a small number of people. Your job is to test the effects of different chemicals on the subjects. Most chemicals have little impact on human beings, but some substances may also cause adverse reactions or even permanent damage.  The chemicals you are testing will form the ingredients of a new AIDS drug, which will bring new hope to millions of AIDS patients around the world. You hope this medicine will save many lives. |  |
| 5.Transplant dilemmas | You are a young doctor working in the emergency surgery ward of your local hospital. Four of the patients under your care in your ward are dying from  grave injury to vital organs as a result of a serious car accident. You have another patient in the ward who is now in good health and ready to be sent  home.  If nothing is done, these four patients are going to die. You can anaesthetize the patient in good health and remove his vital organs to transplant them in  the patients who are dying. You know that this patient will die, but the other four can be saved. |  |
| 6.Border Crossing dilemmas | You are a soldier guarding a border checkpoint between your nation and one troubled by violence and terrorism. You notice a young man in a cheap car approaching the checkpoint with a serious look on his face. You suspect he means to bomb the checkpoint, killing all the soldiers inside. He is quickly approaching your station. |  |
| 7. Trolley dilemmas | You are the driver of a runaway trolley approaching a fork in the tracks. On the tracks going to the left is a group of five railway workers. On the tracks going to the right is a single railway worker.  If you do nothing, the trolley will go to the left, causing the five workers to die. The only way to avoid the deaths of these five workers is to hit a switch on your dashboard that will make the trolley go to the right, leading to the death of the single worker. |  |

## Text S3: Gender differences in depressive levels, alexithymia trait, and utilitarian judgment (1 = male, 2 = female)

| Independent Samples T-Test | | | | | | |
| --- | --- | --- | --- | --- | --- | --- |
|  | *t* | | *df* | *p* | Cohen's *d* | |
| Alexithymia | -0.213 |  | 1773 | 0.832 | -0.010 |  |
| Depressive level | 0.635 |  | 1773 | 0.526 | 0.030 |  |
| Utilitarian judgment | 3.660 |  | 1773 | <.001 | 0.174 |  |
|  | | | | | | |
| Note.  Student's t-test. | | | | | | |

### Descriptives

| Group Descriptives | | | | | |
| --- | --- | --- | --- | --- | --- |
|  | Group | *N* | Mean | SD | SE |
| Alexithymia | male | 953 | 51.949 | 11.766 | 0.381 |
|  | female | 822 | 52.063 | 10.800 | 0.377 |
| Depressive level | male | 953 | 13.910 | 4.560 | 0.148 |
|  | female | 822 | 13.776 | 4.257 | 0.148 |
| Utilitarian judgment | male | 953 | 0.322 | 0.242 | 0.008 |
|  | female | 822 | 0.279 | 0.245 | 0.009 |
|  | | | | | |

## Text S4: Linear Regression among depressive levels, alexithymia trait, and utilitarian judgment

## Linear Regression

| **Model Summary -** Utilitarian judgment | | | | | | | |
| --- | --- | --- | --- | --- | --- | --- | --- |
|  | | | | | Durbin-Watson | | |
| Model | *R* | *R*² | Adjusted *R*² | RMSE | Autocorrelation | Statistic | *p* |
| 1 | 0.108 | 0.012 | 0.010 | 0.243 | -0.001 | 2.001 | 0.982 |
| 2 | 0.105 | 0.011 | 0.010 | 0.243 | 8.245 | 1.997 | 0.953 |
|  | | | | | | | |

| ANOVA | | | | | | |
| --- | --- | --- | --- | --- | --- | --- |
| Model |  | Sum of Squares | *df* | Mean Square | *F* | *p* |
| 1 | Regression | 1.224 | 2 | 0.612 | 10.376 | < .001 |
|  | Residual | 104.517 | 1772 | 0.059 |  | |
|  | Total | 105.741 | 1774 |  |  | |
| 2 | Regression | 1.160 | 1 | 1.160 | 19.672 | < .001 |
|  | Residual | 104.581 | 1773 | 0.059 |  | |
|  | Total | 105.741 | 1774 |  |  |  |
|  | | | | | | |

| Coefficients | | | | | | |
| --- | --- | --- | --- | --- | --- | --- |
| Model |  | Unstandardized | Standard Error | Standardized | *t* | *p* |
| 1 | (Intercept) | 0.201 | 0.028 |  | 7.316 | <.001 |
|  | Alexithymia | 6.223 | 5.989 | 0.029 | 1.039 | 0.299 |
|  | Depressive level | 0.005 | 0.002 | 0.090 | 3.223 | 0.001 |
| 2 | (Intercept) | 0.222 | 0.019 |  | 11.716 | <.001 |
|  | Depressive level | 0.006 | 0.001 | 0.105 | 4.435 | <.001 |
|  | | | | | | |

| Descriptives | | | | |
| --- | --- | --- | --- | --- |
|  | *N* | Mean | SD | SE |
| Utilitarian judgment | 1775 | 0.302 | 0.244 | 0.006 |
| Alexithymia | 1775 | 52.002 | 11.326 | 0.269 |
| Depressive level | 1775 | 13.848 | 4.421 | 0.105 |
|  | | | | |

| **Residuals Statistics** | | | | | | | |
| --- | --- | --- | --- | --- | --- | --- | --- |
|  | **Minimum** | **Maximum** | **Mean** | **SD** | | ***N*** | |
| Predicted Value | 0.274 | 0.430 | 0.302 | 0.026 |  | 1775 |  |
| Residual | -0.396 | 0.674 | 5.536 | 0.243 |  | 1775 |  |
| Std. Predicted Value | -1.096 | 5.010 | 1.063 | 1.000 |  | 1775 |  |
| Std. Residual | -1.636 | 2.776 | 9.007 | 1.000 |  | 1775 |  |
|  | | | | | | | |

### Q-Q Plot Standardized Residuals


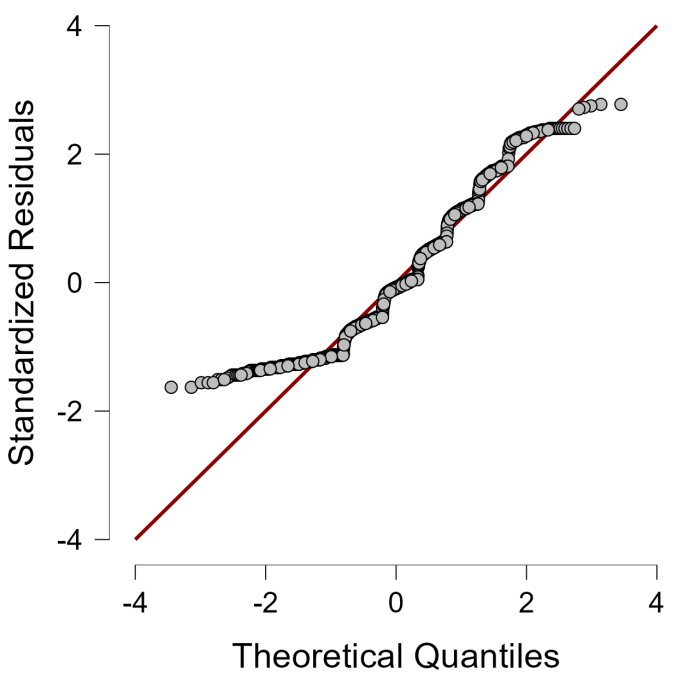


**Text S5:Experiment 2 Process Dissociation Scenarios**

| Moral dilemma task | Congruent/  Incongruent | Text description | Questions |
| --- | --- | --- | --- |
| 1.Footbridge dilemmas | Incongruent | An uncontrol tram was running along the track to five workers working on the track. If the tram was allowed to continue, the five workers would be killed. You are standing on the footbridge above the railway track. The footbridge is between the moving tram and the five workers. As it happens, there is a huge stranger next to you. The only way to save the livess of the five workers is to push the stranger down from the overpass and let him fall into the middle of the track, so as to stop the progress of the tram.  If you do, the stranger will die, but the five workers will be saved. | 1.Would you (nature of action) in order to (outcome of the proposed action)?  A. acceptable  B. unacceptable  2.How moral acceptance is it for you to [nature of action] in order to [outcome of action] |
|  | Congruent | A runaway tram is running along the track to a worker who is working on the track. If the tram is allowed to continue, the worker will be killed. You are standing on the footbridge above the railway track, which is between the moving tram and the worker. As it happens, there is a huge stranger next to you. The only way to save the worker's life is to push the stranger down from the overpass and let him fall into the middle of the track, so as to stop the tram.  If you do, the stranger will die, but the worker will survive. |  |
| 2.Fumes dilemmas | Incongruent | You are the night duty watchman of the hospital. As a result of an accident, deadly smoke entered the hospital's ventilation system. There are three patients  in one room. In another room there is a single patient. If you do nothing, the smoke will spread to the room containing the three patients and cause their death. The only way to avoid their death was to press the control switch, which makes the smoke bypass the rooms containing the three patients.  If you done, smoke will enter the containing one single patient, causing his death, the three patients will be saved. |  |
|  | Congruent | You are the night duty officer of the hospital. As a result of an accident in the next building, deadly smoke entered the hospital's ventilation system. There are three patients in one ward and seven patients in the other. If no measures are taken, the smoke will spread to the room where the three patients live and cause them to suffocate. The only way to avoid their death was to press a control switch to make the smoke bypass the rooms of the three patients.  If this is done, smoke will enter the room with seven patients, causing them to suffocate. |  |
| 3.Car accident dilemmas | Incongruent | You are driving on a busy city street when a young mother with a child falls in front of your car. At this time, it is too late to brake. Your only hope to avoid an accident is to change the driving direction. Unfortunately, the only place you can turn is a small old lady.  If you change your driving direction to avoid young mother and children, you will seriously crash and even kill the old lady. |  |
|  | Congruent | You are driving through a busy city street when all of a sudden a young mother carrying a child trips and falls in front of your car. You are going too fast to stop in time; your only hope is to move out of the way. Unfortunately, there is a group of children walking in the only place you can move to.  If you move out of the way to avoid the young mother and baby, you will seriously injure or kill several of the children. |  |
| 4.Human Research dilemmas | Incongruent | You have been hired by a medical company to conduct research on their drugs. Since drugs must be safe for humans, they are first tried out on humans. Your job is to find out how various chemicals affect humans. Most chemicals have only minor effects on the humans, but some cause them pain or even permanent damage. The chemicals you are researching are supposed to be part of a new AIDS drug cocktail that will give new hope to millions of AIDS victims around the world.  You anticipate saving many lives with the chemicals. |  |
|  | Congruent | You have been hired by a medicine company to conduct research on their drugs. Since drugs must be safe for humans, they are first tried out on humans. Your job is to find out how various chemicals affect humans. Most chemicals have only minor effects on the humans, but some cause them pain or even permanent damage. The chemicals you are researching are supposed to be part of a new acne facial cleanser that will give new hope to people with pimples and greasy skin.  You anticipate making many people feel better about their appearance with the chemicals. |  |
| 5.Transplant dilemmas | Incongruent | You are a doctor. You have five patients. Each of them faced death from organ failure. You have another young patient who is healthy. The only way to save the five patients is to transplant the five organs of the young patient (against his will) to the previous five patients. If you do this, the young man will die, but the other five patients will survive. |  |
|  | Congruent | You are a doctor. You have a patient who is facing death from organ failure. You have another patient. He is healthy. The only way to save the former patient is to transplant an organ of the young patient (against his will) to the former patient.  If you do this, the young man will die, but the former patient will survive. |  |
| 6.Border Crossing dilemmas | Incongruent | You are a soldier guarding a border checkpoint between your nation and one troubled by violence and terrorism. You notice a young man in a cheap car approaching the checkpoint with a serious look on his face. You suspect he means to bomb the checkpoint, killing all the soldiers inside. He is quickly approaching your station. In order to prevent him from approaching, you are going to shoot the man who is approaching the border station.  If you do this, the car will stop, but the young man will die. |  |
|  | Congruent | You are a soldier guarding a border checkpoint between your nation and one troubled by violence and terrorism. You notice a young man in a cheap car approaching the checkpoint with a serious look on his face. You suspect he means to cross the border in order to work illegally inside your country. He is  quickly approaching your station. In order to prevent him from approaching, you are going to shoot the man who is approaching the border station.  If you do this, the car will stop, but the young man will die. |  |
| 7. Trolley dilemmas | Incongruent | You are the driver of a runaway trolley approaching a fork in the tracks. On the tracks going to the left is a group of five railway workers. On the tracks going to the right is a single railway worker.  If you do nothing, the trolley will go to the left, causing the five workers to die. The only way to avoid the deaths of these five workers is to hit a switch on your dashboard that will make the trolley go to the right, leading to the death of the single worker. |  |
|  | Congruent | You are driving an out of control tram, which is moving towards the fork of the track at a very fast speed. There are five railway workers on the left track of the turnout and seven on the right track. If you do not take measures, the tram will continue to drive to the left track, resulting in the death of five workers. The only way to avoid the death of five workers is to press the steering switch on the dashboard to make the tram run to the right track, but this will cause the death of seven workers. |  |

Results

Repeated Measures ANOVA

| Within Subjects Effects | | | | | | | | | | | | | | | |
| --- | --- | --- | --- | --- | --- | --- | --- | --- | --- | --- | --- | --- | --- | --- | --- |
| Cases | | Sum of Squares | | df | | Mean Square | | F | | p | | η² | | η² p | |
| RM Parameter |  | 13.851 |  | 1 |  | 13.851 |  | 251.633 |  | < .001 |  | 0.628 |  | 0.787 |  |
| RM Group ✻ Parameter |  | 0.247 |  | 1 |  | 0.247 |  | 4.491 |  | 0.038 |  | 0.011 |  | 0.062 |  |
| Residuals |  | 3.743 |  | 68 |  | 0.055 |  |  |  |  |  |  |  |  |  |
|  | | | | | | | | | | | | | | | |
| Note.  Type III Sum of Squares | | | | | | | | | | | | | | | |

| Between Subjects Effects | | | | | | | | | | | | | | | |
| --- | --- | --- | --- | --- | --- | --- | --- | --- | --- | --- | --- | --- | --- | --- | --- |
| Cases | | Sum of Squares | | *df* | | Mean Square | | *F* | | *p* | | *η*² | | *η*² _p_ | |
| Group |  | 0.160 |  | 1 |  | 0.160 |  | 2.681 |  | 0.106 |  | 0.007 |  | 0.038 |  |
| Residuals |  | 4.071 |  | 68 |  | 0.060 |  |  |  |  |  |  |  |  |  |
|  | | | | | | | | | | | | | | | |
| Note.  Type III Sum of Squares | | | | | | | | | | | | | | | |

Descriptives

| Descriptives | | | | | | | | | |
| --- | --- | --- | --- | --- | --- | --- | --- | --- | --- |
| RM Factor 1 | | group | | Mean | | SD | | *N* | |
| Parameter *U* |  | DE group |  | 0.143 |  | 0.257 |  | 35 |  |
|  |  | HC group |  | 0.127 |  | 0.158 |  | 35 |  |
| Parameter *D* |  | DE group |  | 0.688 |  | 0.287 |  | 35 |  |
|  |  | HC group |  | 0.840 |  | 0.238 |  | 35 |  |
|  | | | | | | | | | |

Post Hoc Tests

| Post Hoc Comparisons - Group ✻ RM Parameter | | | | | | | | | |
| --- | --- | --- | --- | --- | --- | --- | --- | --- | --- |
|  | |  | | Mean Difference | | SE | | *t* | |
| DE group, Parameter *U* |  | HC group , Parameter *U* |  | 0.016 |  | 0.051 |  | 0.320 |  |
|  |  | DE group, Parameter *D* |  | -0.545 |  | 0.056 |  | -9.718 |  |
|  |  | HC group , Parameter *D* |  | -0.697 |  | 0.057 |  | -12.161 |  |
| HC group , Parameter *U* |  | DE group, Parameter *D* |  | -0.561 |  | 0.057 |  | -9.797 |  |
|  |  | HC group , Parameter *D* |  | -0.713 |  | 0.056 |  | -12.715 |  |
| DE group, Parameter *D* |  | HC group , Parameter *D* |  | -0.152 |  | 0.063 |  | -2.409 |  |
|  | | | | | | | | | |
| Note.  P-value adjusted for comparing a family of 6 | | | | | | | | | |

| Post Hoc Comparisons - Group | | | | | | | | | | | |
| --- | --- | --- | --- | --- | --- | --- | --- | --- | --- | --- | --- |
|  | |  | | Mean Difference | | SE | | *t* | | Cohen's *d* | |
| DE group |  | HC group |  | -0.068 |  | 0.041 |  | -1.637 |  | -0.196 |  |
|  | | | | | | | | | | | |
| Note.  Cohen's d does not correct for multiple comparisons. | | | | | | | | | | | |
