# Supplementary material for: Effect of subclinical depression on moral judgment dilemmas: a process dissociation approach
Source: Sci Rep. 2022 Nov 21;12:20065. doi: 10.1038/s41598-022-24473-2 (PMC9681861; doi:10.1038/s41598-022-24473-2)
Supplement: Supplementary file 2 — Supplementary Information 2. [file 41598_2022_24473_MOESM2_ESM.pdf]

Num	Sex	ale	dep	U-Judgment
1	1	43	7	0.571428571
2	2	32	10	0
3	2	79	11	0
4	2	60	10	0
5	1	20	1	0.571428571
6	1	20	4	0.285714286
7	2	59	8	0.428571429
8	1	20	2	0.142857143
9	1	57	8	0.714285714
10	2	56	4	0.571428571
11	2	51	4	0.857142857
12	2	35	7	0
13	2	56	7	0
14	1	20	0	0.142857143
15	2	28	0	0.285714286
16	2	59	5	0.285714286
17	2	47	7	0.285714286
18	2	62	20	0.571428571
19	2	53	3	0.857142857
20	2	60	12	0.142857143
21	2	62	6	0.142857143
22	2	60	4	0.285714286
23	2	54	3	0
24	2	34	0	0.142857143
25	2	59	7	0
26	1	21	2	0.142857143
27	1	79	16	0.428571429
28	2	29	1	0.428571429
29	2	42	2	0.714285714
30	2	56	1	0.285714286
31	2	59	7	0.428571429
32	2	76	6	0.142857143
33	2	51	4	0
34	2	53	5	0.571428571
35	1	49	5	0
36	2	60	11	0.285714286
37	2	55	4	0
38	1	55	5	0.428571429
39	1	49	10	0.285714286
40	2	53	7	0.571428571
41	2	60	7	0.428571429
42	2	69	11	0.428571429
43	2	39	3	0
44	2	56	6	0.285714286
45	2	53	3	0.285714286
46	2	53	6	0
47	2	60	8	0
48	1	56	3	0.285714286
49	2	49	3	0.285714286
50	2	60	7	0.285714286
51	2	55	3	0.428571429
52	2	26	1	0.285714286
53	1	60	8	0

54	2	62	9	0.142857143
55	1	61	6	0.142857143
56	1	77	9	0.142857143
57	2	62	7	0
58	2	61	4	0.571428571
59	2	78	8	0.142857143
60	2	57	5	0.142857143
61	2	20	3	0
62	2	62	10	0
63	1	55	0	0.142857143
64	1	71	9	0.714285714
65	2	59	5	0.142857143
66	2	51	5	0.571428571
67	2	47	4	0
68	2	53	0	0.285714286
69	1	57	3	0.285714286
70	2	54	4	0
71	1	45	1	0.428571429
72	2	57	6	0.857142857
73	2	61	8	0.571428571
74	2	58	6	0.142857143
75	2	49	3	0.142857143
76	2	23	0	0.142857143
77	2	60	4	0.285714286
78	1	65	9	0.714285714
79	2	77	16	0.428571429
80	2	67	4	0
81	2	55	6	0.428571429
82	1	47	2	0.428571429
83	2	64	1	0.428571429
84	2	55	1	0.428571429
85	2	53	9	0.428571429
86	2	60	10	0.285714286
87	2	44	0	0.714285714
88	2	54	4	0.285714286
89	2	48	0	0.428571429
90	2	67	10	0.714285714
91	2	55	13	0
92	2	48	5	0
93	2	59	5	0.714285714
94	2	55	4	0.571428571
95	2	52	2	0.285714286
96	1	40	2	0.428571429
97	2	57	6	0.428571429
98	2	53	2	0.142857143
99	2	75	8	0.428571429
100	2	62	3	0
101	2	46	0	0.285714286
102	2	62	6	0.142857143
103	1	38	2	0.142857143
104	2	56	9	0.285714286
105	2	49	4	0.142857143
106	2	55	9	0.714285714
107	1	56	6	0.857142857

108	2	45	0	0
109	2	66	24	0.142857143
110	2	65	10	0.285714286
111	2	56	5	0.285714286
112	2	51	7	0
113	2	65	6	0.714285714
114	2	58	6	0
115	2	54	4	0.142857143
116	2	56	7	0.285714286
117	2	54	7	0.285714286
118	1	52	6	0.285714286
119	2	36	1	0.142857143
120	2	50	6	0.571428571
121	2	50	4	0
122	1	58	1	0.285714286
123	1	69	17	0.571428571
124	2	56	1	0.142857143
125	1	62	6	0.571428571
126	2	60	4	0
127	2	61	4	0
128	1	54	1	0.285714286
129	1	58	8	0.285714286
130	2	56	3	0
131	2	59	6	0
132	1	50	2	0.571428571
133	2	54	4	0.285714286
134	2	48	10	0.571428571
135	2	58	5	0.285714286
136	2	64	7	0.714285714
137	2	54	1	0.714285714
138	2	56	8	0.142857143
139	1	39	0	0.142857143
140	2	52	5	0.428571429
141	2	47	3	0.285714286
142	2	53	6	0
143	2	49	1	0.285714286
144	2	62	8	0
145	2	55	8	0.571428571
146	1	71	14	0.428571429
147	1	70	13	0.285714286
148	1	56	21	0
149	2	71	6	0.285714286
150	2	45	6	0.857142857
151	2	57	10	0.142857143
152	1	62	12	0.285714286
153	1	84	21	0
154	2	20	3	0.142857143
155	1	60	0	0.428571429
156	1	64	6	0.428571429
157	1	49	6	0.285714286
158	1	46	0	0.571428571
159	1	66	10	0.428571429
160	1	37	1	0.285714286
161	1	49	8	0.285714286

162	1	64	7	0.428571429
163	1	43	0	0
164	1	54	8	0.142857143
165	1	47	3	0
166	2	83	22	0.428571429
167	1	71	7	0.142857143
168	1	55	5	0.142857143
169	1	43	2	0.142857143
170	1	64	7	0
171	1	56	4	0.285714286
172	1	32	0	0.428571429
173	1	53	5	0.571428571
174	1	49	0	0.142857143
175	1	59	5	0.285714286
176	1	46	6	0.285714286
177	1	54	6	0.142857143
178	1	66	5	0.714285714
179	1	52	5	0.142857143
180	1	49	2	0.142857143
181	1	57	6	0.285714286
182	1	63	6	0.428571429
183	1	39	0	0.142857143
184	1	61	3	0.428571429
185	1	54	10	0.285714286
186	1	62	7	0.142857143
187	1	36	1	0.571428571
188	1	66	4	0.714285714
189	1	54	2	0.142857143
190	1	53	0	0.285714286
191	1	50	4	0.428571429
192	1	44	4	0.571428571
193	1	43	6	0.571428571
194	1	28	6	0.285714286
195	1	60	3	0.428571429
196	1	49	0	0.142857143
197	1	66	8	0.285714286
198	1	44	3	0.714285714
199	1	64	9	0.571428571
200	2	60	3	0.428571429
201	2	58	8	0.285714286
202	1	43	1	0.428571429
203	1	69	10	0.857142857
204	1	34	0	0.714285714
205	1	52	2	0.285714286
206	1	55	5	0.428571429
207	1	69	9	0.285714286
208	1	56	8	0.142857143
209	1	52	4	0.428571429
210	1	62	5	0
211	1	53	2	0
212	1	48	2	0.285714286
213	1	58	6	0.142857143
214	1	71	5	0.142857143
215	1	57	12	0.571428571

216	1	55	1	0.285714286
217	1	57	1	0.142857143
218	1	54	2	0.285714286
219	1	49	2	0.142857143
220	1	46	3	0.714285714
221	1	67	11	0.571428571
222	1	64	7	0
223	1	44	0	0.285714286
224	1	40	2	0.285714286
225	1	57	3	0.428571429
226	1	52	1	0.285714286
227	1	50	1	0
228	1	66	7	0.142857143
229	1	61	7	0
230	1	49	2	0.285714286
231	1	50	6	0.285714286
232	1	63	2	0
233	1	34	1	0.142857143
234	1	54	4	0.428571429
235	1	67	6	0.285714286
236	1	52	1	0.571428571
237	1	44	0	0.142857143
238	1	53	9	1
239	1	55	5	0.142857143
240	1	51	0	0.285714286
241	1	44	3	0.285714286
242	1	56	3	0.571428571
243	1	56	1	0
244	1	38	3	0.428571429
245	1	57	4	0.285714286
246	1	54	6	0.285714286
247	1	71	14	0.571428571
248	1	58	2	0.714285714
249	1	69	7	0.571428571
250	1	48	1	0.571428571
251	1	66	9	0.428571429
252	1	54	7	0
253	1	60	2	0.571428571
254	1	61	3	0.285714286
255	1	56	5	0.714285714
256	2	46	5	0.428571429
257	1	45	1	0
258	2	41	0	0
259	2	34	4	0.571428571
260	2	47	3	0.142857143
261	2	20	0	0.571428571
262	2	29	4	0.142857143
263	1	41	1	0.142857143
264	2	42	0	0
265	2	60	8	0
266	2	60	15	0.714285714
267	2	43	1	0.142857143
268	2	45	6	0.142857143
269	2	70	11	0.571428571

270	2	20	0	0.285714286
271	2	23	3	0.571428571
272	2	55	0	0
273	2	55	13	0.142857143
274	2	27	1	0
275	2	58	4	0
276	1	44	12	0.714285714
277	2	31	2	0
278	2	50	6	0.142857143
279	2	47	2	0.285714286
280	2	52	5	0
281	2	48	0	0.428571429
282	2	45	1	0
283	2	55	0	0.285714286
284	2	29	4	0.142857143
285	2	27	2	0
286	2	53	8	0.142857143
287	2	20	0	0.428571429
288	1	53	7	0
289	1	72	0	0.142857143
290	2	65	7	0.428571429
291	2	72	11	0.142857143
292	2	49	8	0.571428571
293	2	45	0	0
294	1	60	6	0
295	1	43	2	0.571428571
296	2	59	5	0
297	2	62	13	0
298	2	50	6	0.285714286
299	2	42	0	0
300	1	43	8	0.714285714
301	2	66	0	0
302	2	57	17	0.428571429
303	2	56	0	0.285714286
304	1	47	13	0.428571429
305	2	53	6	0.428571429
306	2	63	8	0
307	2	38	1	0.142857143
308	2	44	2	0
309	1	60	8	0.142857143
310	2	42	3	0
311	2	60	11	0.285714286
312	2	20	0	0
313	2	45	2	0
314	2	20	1	0
315	1	53	0	0.142857143
316	2	40	5	0
317	2	45	5	0.857142857
318	2	42	0	0.285714286
319	2	57	3	0
320	2	20	0	0
321	2	41	6	0.428571429
322	2	53	2	0.428571429
323	2	47	4	0.142857143

324	1	40	0	0
325	2	41	4	0.142857143
326	2	52	3	0
327	2	61	7	0.857142857
328	2	46	2	0.428571429
329	2	31	5	0.142857143
330	1	43	0	0.142857143
331	2	42	0	0.142857143
332	2	54	4	0.285714286
333	2	44	3	0
334	2	69	8	0.714285714
335	1	40	0	0.142857143
336	2	56	6	0
337	2	48	2	0
338	1	59	10	0.428571429
339	2	36	0	0
340	2	45	7	0.142857143
341	2	60	7	0.285714286
342	2	65	8	0.428571429
343	2	53	0	0.714285714
344	2	39	4	0
345	2	59	8	0
346	2	63	4	0.428571429
347	2	46	3	0.285714286
348	2	61	4	0.285714286
349	1	53	0	0.571428571
350	2	58	8	0
351	2	55	10	0.285714286
352	2	61	3	0.142857143
353	2	59	6	0.285714286
354	2	31	0	0.285714286
355	2	56	1	0.285714286
356	2	55	0	0.285714286
357	2	49	0	0
358	2	49	3	0.428571429
359	1	39	0	0.571428571
360	1	51	7	0.142857143
361	1	26	0	0
362	2	39	0	0
363	2	50	3	0.571428571
364	2	40	0	0.142857143
365	1	65	9	0.285714286
366	2	36	0	0.142857143
367	2	49	0	0.571428571
368	1	51	16	0
369	2	58	5	0
370	2	40	0	0
371	1	60	6	0.142857143
372	2	54	6	0
373	2	50	7	0.428571429
374	2	42	0	0
375	2	56	8	0.142857143
376	2	54	3	0.285714286
377	2	57	8	0

378	2	43	5	0.857142857
379	2	45	0	0.285714286
380	1	55	2	0.428571429
381	2	58	6	0.857142857
382	2	53	4	0.428571429
383	2	51	3	0.571428571
384	1	43	0	0
385	2	65	5	0.285714286
386	2	59	2	0
387	2	56	7	0.285714286
388	1	49	0	0.428571429
389	1	49	0	0.857142857
390	1	61	16	0
391	2	54	2	0
392	2	55	0	0
393	2	55	0	0.428571429
394	1	66	11	0.142857143
395	1	60	4	0.285714286
396	1	53	7	0.714285714
397	2	46	0	0.428571429
398	2	52	4	0
399	2	63	8	0.428571429
400	2	51	1	0.428571429
401	2	52	5	0.142857143
402	2	51	0	0.142857143
403	2	51	2	0.857142857
404	2	70	9	0
405	1	48	2	0
406	2	54	10	0.142857143
407	2	64	19	0.142857143
408	2	53	2	0.142857143
409	2	77	6	0.571428571
410	2	61	14	0.285714286
411	2	48	2	0.142857143
412	2	62	2	0.142857143
413	2	54	5	0.428571429
414	2	44	10	0.571428571
415	2	51	6	0
416	2	46	6	0
417	2	51	2	0.428571429
418	1	44	1	0
419	2	56	1	0
420	2	52	2	0.571428571
421	2	50	3	0.285714286
422	2	57	2	0.714285714
423	1	57	15	0.142857143
424	1	52	3	0.285714286
425	2	56	2	0.285714286
426	2	64	8	0.428571429
427	1	60	8	0
428	1	21	0	0.714285714
429	1	60	9	0
430	1	28	0	0
431	1	53	0	0.142857143

432	1	65	8	0.285714286
433	1	24	0	0
434	1	36	4	0
435	1	60	9	0.857142857
436	2	24	0	0
437	1	63	8	0.571428571
438	1	20	0	0.714285714
439	1	60	0	0.285714286
440	1	20	0	0
441	1	26	0	0
442	1	41	0	0.571428571
443	1	39	1	0.428571429
444	1	68	16	0.428571429
445	1	28	0	0.142857143
446	1	78	15	0.571428571
447	1	65	2	0.428571429
448	1	29	0	0.714285714
449	1	60	0	0.142857143
450	1	52	10	0.571428571
451	1	56	4	0.285714286
452	1	60	7	0.857142857
453	1	62	5	0.285714286
454	1	60	8	0.714285714
455	1	57	8	0.142857143
456	1	61	13	0.857142857
457	1	20	3	0
458	2	52	7	0
459	1	56	0	0
460	1	59	9	0.142857143
461	1	60	8	0.428571429
462	2	58	6	0.285714286
463	2	29	4	0.428571429
464	1	44	0	0.285714286
465	1	22	2	0.857142857
466	1	60	10	0.571428571
467	1	58	5	0.142857143
468	1	71	10	0.428571429
469	1	33	0	0.857142857
470	1	52	0	0.571428571
471	1	70	9	0.428571429
472	1	55	3	0
473	1	53	2	0.142857143
474	1	42	0	0
475	2	46	0	0
476	1	57	8	0.285714286
477	1	55	10	0.142857143
478	1	46	4	0.142857143
479	1	48	5	0.142857143
480	1	24	0	0.571428571
481	1	49	1	0
482	2	44	0	0
483	2	46	0	0.714285714
484	1	49	2	0.571428571
485	2	50	7	0.285714286

486	1	51	2	0.428571429
487	1	48	2	0.428571429
488	1	40	11	0.142857143
489	1	63	0	0.714285714
490	1	64	5	0.285714286
491	1	69	5	0
492	1	49	0	0.285714286
493	1	55	5	0.428571429
494	1	56	6	0
495	1	41	2	0.571428571
496	2	54	1	0.571428571
497	1	58	4	0.428571429
498	2	49	3	0.142857143
499	1	43	0	0.714285714
500	1	60	4	0.428571429
501	1	56	5	0.285714286
502	1	50	0	0.285714286
503	1	49	0	0.285714286
504	2	43	1	0.285714286
505	1	56	4	0.428571429
506	2	46	4	0
507	1	62	8	0.142857143
508	2	33	2	0.428571429
509	1	48	4	0.571428571
510	1	47	1	0.142857143
511	2	36	0	0.428571429
512	1	51	9	0.285714286
513	1	45	4	0.285714286
514	1	56	6	0.285714286
515	1	49	3	0.571428571
516	1	54	4	0.142857143
517	2	52	3	0.142857143
518	1	47	6	0.571428571
519	1	46	9	0.571428571
520	1	48	1	0
521	2	47	4	0.428571429
522	1	67	5	0.285714286
523	1	50	2	0.285714286
524	1	55	2	0.428571429
525	2	51	4	0
526	1	48	2	0.142857143
527	1	57	0	0.714285714
528	1	58	1	0.571428571
529	1	57	2	0
530	1	50	1	0.142857143
531	1	27	0	0.571428571
532	2	52	2	0
533	1	51	2	0.714285714
534	1	57	5	0.571428571
535	1	52	3	0.428571429
536	1	58	9	0.142857143
537	1	47	5	0
538	2	64	1	0.571428571
539	1	65	3	0.571428571

540	2	61	3	0.285714286
541	1	56	5	0.428571429
542	1	60	8	0.285714286
543	2	58	5	0.142857143
544	2	52	3	0.714285714
545	2	30	0	0.285714286
546	1	58	2	0.285714286
547	1	49	3	0.714285714
548	1	36	2	0.142857143
549	1	34	1	0.571428571
550	1	38	0	0.142857143
551	1	58	26	0.285714286
552	2	50	0	0.571428571
553	1	56	7	0.428571429
554	1	52	2	0
555	1	47	1	0.428571429
556	2	49	2	0.714285714
557	1	68	5	0.571428571
558	1	47	8	0.714285714
559	1	54	1	0.428571429
560	1	61	2	0.714285714
561	2	50	3	0.428571429
562	1	53	0	0.285714286
563	1	60	3	0
564	1	57	6	0.571428571
565	1	42	7	0.571428571
566	2	59	8	0
567	1	55	5	0.285714286
568	1	47	0	0
569	1	49	6	0.857142857
570	1	57	2	0
571	1	46	0	0.857142857
572	1	50	4	0.142857143
573	1	53	1	0.428571429
574	1	53	0	0.142857143
575	1	55	7	0.428571429
576	1	53	4	0.571428571
577	2	53	3	0.571428571
578	1	51	7	0.285714286
579	2	39	5	0.571428571
580	1	58	7	0.571428571
581	2	61	3	0.428571429
582	1	54	3	0.428571429
583	1	46	3	0.571428571
584	1	63	7	0.142857143
585	1	47	0	0.142857143
586	1	59	6	0.428571429
587	1	33	4	0.714285714
588	1	41	0	0
589	1	40	0	0.285714286
590	1	61	7	0.428571429
591	2	54	5	0.428571429
592	1	55	7	0.285714286
593	1	44	5	0.428571429

594	1	52	0	0.857142857
595	1	59	11	0.428571429
596	1	53	4	0.714285714
597	1	46	4	0.285714286
598	2	55	0	0.428571429
599	1	51	1	0.857142857
600	1	44	6	0.571428571
601	1	66	3	0
602	1	51	2	0.142857143
603	1	49	5	0.285714286
604	1	54	4	0.571428571
605	2	55	5	0
606	1	59	9	0.142857143
607	1	45	3	0.285714286
608	1	44	1	0
609	1	56	2	0.571428571
610	1	59	2	0.571428571
611	1	40	4	0.142857143
612	1	56	6	0.428571429
613	1	44	3	0.571428571
614	1	57	7	0.428571429
615	1	62	3	0.142857143
616	1	55	1	0.285714286
617	1	32	2	0.142857143
618	1	40	1	0.428571429
619	1	66	1	0.714285714
620	1	59	3	0.285714286
621	1	53	3	0.142857143
622	1	55	2	0.142857143
623	1	47	4	0.142857143
624	1	50	2	0
625	2	47	0	0
626	1	57	2	0.428571429
627	1	48	0	0.142857143
628	1	56	0	0.285714286
629	1	49	0	0.285714286
630	1	51	0	0
631	2	60	8	0
632	1	55	9	0.857142857
633	1	34	2	0.285714286
634	1	62	17	0.285714286
635	1	28	0	0.714285714
636	1	96	24	0.714285714
637	1	91	27	1
638	2	54	9	0
639	2	40	0	0.142857143
640	1	27	0	0.285714286
641	1	48	10	0.142857143
642	1	32	2	0.142857143
643	2	34	1	0
644	2	26	0	0.571428571
645	1	60	12	0.428571429
646	2	58	8	0.428571429
647	1	26	0	0

648	1	20	1	0.285714286
649	1	38	2	0
650	1	43	4	0.857142857
651	2	60	9	0
652	1	60	0	0
653	2	52	2	0.571428571
654	2	50	5	0.285714286
655	1	29	0	0.285714286
656	1	49	16	0.857142857
657	2	49	0	0
658	1	40	9	0.428571429
659	2	22	5	0.142857143
660	2	55	2	0
661	1	30	4	0
662	2	60	10	0.142857143
663	2	60	9	0
664	1	22	0	0
665	1	32	3	0.571428571
666	1	67	4	0.285714286
667	1	30	9	0.714285714
668	1	57	13	0.571428571
669	1	56	1	0.285714286
670	2	58	0	0.142857143
671	1	52	0	0.571428571
672	1	35	2	0.285714286
673	2	56	6	0.428571429
674	2	50	3	0.285714286
675	2	60	9	0
676	1	58	9	0.857142857
677	2	60	7	0
678	2	41	3	0.285714286
679	2	26	3	0
680	1	28	6	0
681	2	56	3	0.428571429
682	1	43	0	0.142857143
683	2	31	1	0.714285714
684	2	25	2	0.428571429
685	2	61	3	0
686	2	34	1	0.571428571
687	2	58	18	0
688	1	66	5	0.142857143
689	1	20	0	0.142857143
690	2	43	0	0.428571429
691	2	59	6	0.285714286
692	2	51	4	0
693	1	42	0	0
694	2	62	6	0
695	2	44	4	0.142857143
696	2	50	3	0
697	1	40	2	0
698	1	51	2	0.428571429
699	1	48	4	0.428571429
700	2	47	2	0.142857143
701	1	25	3	0.571428571

702	1	62	8	0.714285714
703	2	52	3	0.428571429
704	1	50	1	0.428571429
705	2	23	3	0.142857143
706	1	64	5	0.142857143
707	2	41	2	0.285714286
708	2	57	6	0
709	1	55	7	0.142857143
710	2	49	17	0.142857143
711	2	20	0	0.285714286
712	1	37	1	0.142857143
713	1	60	17	0.714285714
714	2	58	10	0.142857143
715	1	44	2	0
716	1	56	10	0.142857143
717	1	60	13	0.857142857
718	1	60	8	0.142857143
719	2	35	1	0
720	1	61	7	0
721	2	84	9	0.571428571
722	1	49	5	0.285714286
723	2	22	0	0
724	1	64	5	0
725	2	60	5	0.285714286
726	2	64	11	0.428571429
727	1	60	8	0.142857143
728	2	59	5	0.571428571
729	1	31	4	0.285714286
730	2	65	15	0.142857143
731	2	56	6	0
732	2	41	0	0
733	2	27	0	0
734	1	33	7	0.857142857
735	2	56	7	0.142857143
736	2	53	5	0.285714286
737	2	50	6	0.285714286
738	1	58	6	0.428571429
739	2	47	3	0.714285714
740	2	54	2	0.285714286
741	2	61	3	0
742	2	63	6	0
743	2	42	5	0.142857143
744	2	60	7	0.428571429
745	1	60	10	1
746	1	22	1	0.285714286
747	2	46	0	0.428571429
748	2	45	0	0
749	2	26	0	0
750	1	44	0	0
751	1	53	10	0.571428571
752	1	21	0	0.285714286
753	2	67	8	0.285714286
754	2	35	1	0.571428571
755	1	21	0	0.571428571

756	2	55	11	0.142857143
757	2	49	1	0.428571429
758	1	49	3	0
759	1	38	0	0.285714286
760	2	29	0	0.571428571
761	1	56	2	0.142857143
762	2	62	7	0.571428571
763	1	54	0	0.142857143
764	2	48	5	0.285714286
765	2	71	10	0.142857143
766	1	60	7	0.285714286
767	2	49	3	0.571428571
768	2	57	9	0.142857143
769	1	29	3	0.142857143
770	1	39	18	0
771	2	55	10	0
772	1	57	0	0.428571429
773	2	50	1	0.142857143
774	2	54	2	0.428571429
775	1	40	0	0.285714286
776	1	34	0	0
777	2	45	1	0.142857143
778	1	28	0	0.285714286
779	2	48	0	0.285714286
780	1	55	5	0.571428571
781	2	62	14	0.142857143
782	2	61	9	0.285714286
783	2	58	5	0.571428571
784	1	54	2	0.571428571
785	1	44	4	0.428571429
786	1	61	3	0.571428571
787	2	62	2	0.428571429
788	2	46	1	0.285714286
789	2	46	4	0.142857143
790	2	55	6	0
791	2	60	8	0.285714286
792	2	49	1	0.142857143
793	1	46	0	0.142857143
794	2	28	1	0.571428571
795	2	61	8	0.142857143
796	2	46	1	0.857142857
797	1	20	1	0.142857143
798	2	61	5	0.714285714
799	2	64	9	0.142857143
800	2	58	2	0.142857143
801	2	58	3	0.285714286
802	1	54	1	0
803	1	53	0	0.428571429
804	2	59	5	0.142857143
805	2	57	6	0.142857143
806	2	50	5	0.428571429
807	1	80	27	1
808	1	40	0	0
809	1	49	0	0.285714286

810	2	29	4	0
811	1	57	10	0.571428571
812	2	59	10	0
813	2	50	2	0.714285714
814	2	53	0	0.571428571
815	1	47	2	0.571428571
816	2	52	5	0.428571429
817	1	20	0	0.571428571
818	2	60	8	0.428571429
819	1	55	0	0
820	1	61	5	0.714285714
821	1	53	5	0.714285714
822	2	44	0	0.142857143
823	1	22	4	0.285714286
824	2	49	1	0
825	2	45	0	0.142857143
826	1	69	9	0.428571429
827	2	47	1	0.285714286
828	1	47	3	0.142857143
829	2	54	3	0.142857143
830	2	65	8	0.571428571
831	2	46	5	0.142857143
832	2	48	3	0.428571429
833	2	55	14	0
834	1	55	10	0.571428571
835	2	41	5	0.428571429
836	2	52	2	0.142857143
837	1	58	6	0.142857143
838	1	51	7	0.142857143
839	2	54	9	0
840	2	51	4	0.285714286
841	2	46	6	0.142857143
842	1	51	0	0.428571429
843	2	51	7	0.285714286
844	2	46	0	0.142857143
845	1	45	4	0.142857143
846	1	56	3	0
847	2	41	1	0
848	2	54	1	0.142857143
849	1	57	3	0.857142857
850	1	47	4	0
851	1	56	4	0.428571429
852	2	59	5	0
853	1	41	4	0.428571429
854	2	61	6	0
855	1	46	1	0.285714286
856	2	74	12	0
857	1	25	0	0.285714286
858	1	55	8	0.714285714
859	2	47	3	0
860	2	53	3	0.142857143
861	2	35	2	0.857142857
862	2	58	8	0.571428571
863	1	60	8	0

864	1	33	11	0
865	2	44	3	0
866	2	50	5	0.428571429
867	2	57	4	0
868	2	48	0	0.285714286
869	1	36	4	0.142857143
870	2	62	5	0.571428571
871	2	75	0	0.142857143
872	2	53	6	0
873	1	53	3	0
874	2	50	6	0.285714286
875	2	56	3	0.571428571
876	2	48	4	0.428571429
877	2	54	9	0.285714286
878	2	52	2	0.285714286
879	2	49	2	0.285714286
880	1	44	3	0
881	1	52	6	0.142857143
882	1	60	9	0.571428571
883	2	44	0	0.142857143
884	2	55	5	0.285714286
885	1	26	0	0
886	2	24	0	0.285714286
887	2	54	6	0
888	2	58	1	0.428571429
889	1	60	7	0.142857143
890	2	52	0	0
891	2	55	10	0
892	2	55	10	0.142857143
893	1	51	3	0.285714286
894	2	47	4	0.142857143
895	1	53	9	0.428571429
896	2	57	4	0.285714286
897	2	70	15	0.428571429
898	2	55	0	0
899	2	57	18	0
900	2	49	1	0.285714286
901	2	29	0	0
902	1	57	8	0.142857143
903	2	48	2	0
904	1	61	8	0.857142857
905	1	74	7	0.714285714
906	1	51	4	0.142857143
907	1	60	15	0.142857143
908	1	52	11	0.142857143
909	1	54	4	0
910	1	48	10	0.714285714
911	1	55	8	0.285714286
912	2	59	8	0.142857143
913	2	49	6	0.142857143
914	1	44	0	0
915	2	54	3	0.285714286
916	2	59	4	0.285714286
917	2	56	2	0.142857143

918	2	49	0	0.571428571
919	1	61	7	0.285714286
920	1	68	4	0.142857143
921	2	56	3	0.142857143
922	2	54	0	0
923	2	45	0	0.142857143
924	2	42	0	0.142857143
925	1	49	2	0.285714286
926	1	49	0	0.285714286
927	2	40	3	0.285714286
928	1	58	6	0
929	2	52	4	0
930	2	32	0	0.285714286
931	1	41	2	0
932	1	75	19	0.714285714
933	1	58	8	0.428571429
934	1	60	7	0.285714286
935	2	45	0	0
936	2	32	7	0.285714286
937	1	59	10	0.571428571
938	2	44	2	0.571428571
939	1	56	7	0.428571429
940	2	62	11	0.142857143
941	1	58	8	0.142857143
942	2	52	8	0.857142857
943	2	61	12	0.428571429
944	2	51	1	0.571428571
945	2	75	7	0.714285714
946	1	51	0	0.285714286
947	1	58	7	0.142857143
948	1	51	3	0.285714286
949	1	36	2	0.428571429
950	1	46	1	0.857142857
951	2	58	8	0.285714286
952	1	38	0	0.428571429
953	1	54	17	0.285714286
954	2	51	7	0.571428571
955	1	47	0	0
956	2	66	15	0
957	2	40	0	0.142857143
958	1	76	8	0.857142857
959	1	57	0	0.857142857
960	1	58	10	0.571428571
961	2	67	6	0
962	2	56	4	0
963	2	55	0	0
964	1	57	4	0.571428571
965	1	46	3	0.571428571
966	2	54	3	0.571428571
967	2	66	7	0.285714286
968	1	60	4	0.142857143
969	2	41	0	0.285714286
970	2	39	0	0.571428571
971	1	76	6	0.285714286

972	1	61	8	0.285714286
973	2	54	8	0
974	2	46	0	0.142857143
975	2	51	2	0.714285714
976	1	53	4	0.142857143
977	2	51	2	0.428571429
978	1	55	3	0.428571429
979	1	50	3	0.285714286
980	1	59	8	0.285714286
981	2	37	0	0.428571429
982	1	57	2	0
983	2	49	1	0.857142857
984	1	57	6	0.142857143
985	2	51	1	0.285714286
986	1	63	12	0.142857143
987	2	69	16	0.428571429
988	1	52	3	0.571428571
989	2	67	6	0
990	2	58	6	0.285714286
991	2	41	1	0.428571429
992	1	50	6	0.285714286
993	2	60	8	0.142857143
994	1	67	13	0.571428571
995	2	69	21	0.285714286
996	2	49	0	0
997	2	60	11	0.142857143
998	2	46	1	0.714285714
999	2	49	4	0.285714286
1000	1	43	0	0.142857143
1001	1	57	4	0.714285714
1002	1	62	9	0.428571429
1003	2	55	15	0.428571429
1004	1	60	8	0
1005	1	60	14	0.142857143
1006	1	58	8	0.285714286
1007	2	57	7	0
1008	2	61	6	0.857142857
1009	2	49	8	0.428571429
1010	2	49	6	0.285714286
1011	2	47	0	0
1012	2	33	0	0.714285714
1013	2	45	6	0.714285714
1014	1	25	2	0
1015	2	50	1	0.142857143
1016	2	43	0	0.142857143
1017	1	51	0	0.285714286
1018	2	20	0	0.142857143
1019	1	57	0	0.142857143
1020	1	65	18	0.285714286
1021	2	41	0	0.857142857
1022	2	74	21	0.142857143
1023	1	36	1	0
1024	1	20	0	0.571428571
1025	1	64	7	0.714285714

1026	1	58	6	0.142857143
1027	2	51	4	0.428571429
1028	1	50	1	0.428571429
1029	1	49	0	0.285714286
1030	1	56	2	0
1031	2	46	5	0.571428571
1032	2	56	8	0.428571429
1033	1	38	1	0
1034	1	51	3	0.571428571
1035	1	48	6	0.428571429
1036	1	44	0	0
1037	2	41	3	0.428571429
1038	1	54	2	0.428571429
1039	2	56	2	0
1040	1	58	2	0.428571429
1041	2	68	8	0.142857143
1042	1	61	3	0.571428571
1043	1	87	12	0.571428571
1044	1	45	1	0
1045	2	33	2	0
1046	2	40	5	0.571428571
1047	1	57	3	0.428571429
1048	1	55	2	0.142857143
1049	1	43	1	0.142857143
1050	2	45	0	0
1051	2	42	0	0.142857143
1052	2	52	1	0.428571429
1053	2	52	6	0.714285714
1054	1	61	8	0.571428571
1055	2	45	5	0.142857143
1056	2	53	5	0
1057	2	59	3	0.571428571
1058	1	64	13	0.428571429
1059	1	58	6	0.142857143
1060	2	62	9	0.142857143
1061	1	55	6	0.285714286
1062	2	55	4	0.285714286
1063	2	75	14	0.428571429
1064	1	50	5	0.142857143
1065	1	59	5	0.571428571
1066	1	70	8	0.428571429
1067	2	45	0	0.142857143
1068	1	48	3	0.142857143
1069	2	58	4	0.285714286
1070	1	56	8	0.285714286
1071	1	62	11	0.428571429
1072	1	53	3	0.285714286
1073	2	58	9	1
1074	1	63	11	0.142857143
1075	2	56	3	0.714285714
1076	1	43	0	0.285714286
1077	1	50	0	0.142857143
1078	1	57	7	0.428571429
1079	2	20	5	0

1080	2	46	3	0.857142857
1081	2	59	13	0.571428571
1082	1	59	14	0.142857143
1083	1	77	13	0
1084	2	59	6	0
1085	2	20	0	0.285714286
1086	1	63	6	0.857142857
1087	1	42	5	0.428571429
1088	1	60	6	0.142857143
1089	2	69	7	0
1090	1	57	6	0.571428571
1091	2	56	8	0.428571429
1092	1	36	0	0.285714286
1093	1	59	1	0.142857143
1094	2	56	12	0
1095	2	50	3	0
1096	1	86	6	0.714285714
1097	1	41	0	0
1098	1	70	7	0.571428571
1099	2	66	18	0.285714286
1100	1	61	7	0.142857143
1101	2	50	6	0.285714286
1102	1	47	3	0.285714286
1103	2	44	4	0.142857143
1104	1	63	10	0.285714286
1105	2	59	3	0.285714286
1106	1	66	10	0
1107	2	51	5	0.428571429
1108	1	52	1	0
1109	2	47	4	0.571428571
1110	1	72	12	0.142857143
1111	2	55	2	0.142857143
1112	2	62	6	0.428571429
1113	1	64	8	0
1114	2	75	6	0
1115	2	53	5	0.142857143
1116	1	50	11	0.142857143
1117	1	49	4	0
1118	2	48	2	0
1119	1	54	2	0.142857143
1120	2	54	3	0.285714286
1121	2	62	12	0.142857143
1122	2	58	6	0.285714286
1123	2	49	12	0
1124	1	50	0	0.285714286
1125	2	30	8	0.285714286
1126	1	56	8	0.428571429
1127	1	20	0	0
1128	2	55	2	0
1129	2	55	0	0.285714286
1130	2	51	0	0.142857143
1131	2	39	4	0.285714286
1132	1	27	6	0.285714286
1133	1	58	15	0.285714286

1134	2	47	2	0.285714286
1135	2	52	4	0.571428571
1136	1	32	2	0.142857143
1137	1	63	9	0.285714286
1138	2	50	5	0
1139	2	49	15	0.714285714
1140	2	58	8	0.142857143
1141	2	57	2	0.714285714
1142	1	82	23	0.285714286
1143	1	54	5	0.571428571
1144	2	55	7	0.428571429
1145	1	54	0	0.142857143
1146	2	38	1	0.285714286
1147	1	52	8	0.285714286
1148	1	50	6	0
1149	2	33	4	0.428571429
1150	1	74	3	0.142857143
1151	2	43	1	0.142857143
1152	2	60	10	0
1153	2	55	2	0
1154	1	52	3	0.285714286
1155	1	57	8	0.428571429
1156	1	47	2	0.285714286
1157	2	34	5	0.571428571
1158	1	51	1	0.142857143
1159	2	53	5	0
1160	2	50	0	0.714285714
1161	1	67	10	0.428571429
1162	1	42	0	0.571428571
1163	2	63	14	0.142857143
1164	1	54	4	0.142857143
1165	1	69	19	0.142857143
1166	1	48	2	0.285714286
1167	1	59	5	0.428571429
1168	1	54	2	0.142857143
1169	2	54	2	0.428571429
1170	2	40	0	0
1171	2	67	12	0.571428571
1172	1	47	2	0.142857143
1173	2	74	13	0.428571429
1174	2	58	8	0.285714286
1175	2	47	2	0.142857143
1176	1	60	7	0.285714286
1177	1	56	12	0.857142857
1178	1	43	6	0.142857143
1179	2	59	5	0.285714286
1180	2	53	8	0.571428571
1181	1	54	9	0.428571429
1182	1	56	3	0.714285714
1183	1	48	3	0
1184	1	56	7	0.571428571
1185	1	52	7	0
1186	1	55	7	0.142857143
1187	2	53	3	0.285714286

1188	2	55	4	0.285714286
1189	2	71	16	0.142857143
1190	1	51	4	0.285714286
1191	1	39	1	0.285714286
1192	1	38	4	0.142857143
1193	1	39	1	0
1194	1	57	2	0.428571429
1195	1	48	8	0
1196	2	67	3	0.714285714
1197	2	50	4	0.428571429
1198	1	52	3	0.714285714
1199	1	63	8	0
1200	2	34	1	0.142857143
1201	2	54	6	0.285714286
1202	1	62	7	0.428571429
1203	2	58	4	0.428571429
1204	2	49	12	0
1205	1	60	9	0.285714286
1206	1	58	6	0
1207	2	41	0	0.285714286
1208	1	37	5	0.428571429
1209	2	65	5	0.285714286
1210	1	55	0	0
1211	2	45	4	0.428571429
1212	1	60	0	0
1213	1	47	7	0.142857143
1214	1	77	15	0.285714286
1215	2	60	9	0.285714286
1216	1	60	1	0.142857143
1217	1	56	5	0
1218	2	40	0	0.428571429
1219	2	59	9	0.285714286
1220	1	49	1	0.571428571
1221	1	55	5	0.285714286
1222	2	60	6	0
1223	2	57	4	0
1224	1	54	0	0.285714286
1225	1	39	9	0
1226	1	53	6	0
1227	2	50	0	0.857142857
1228	1	54	8	0.571428571
1229	2	56	0	0.714285714
1230	1	57	4	0.142857143
1231	2	43	2	0.285714286
1232	1	60	0	0.142857143
1233	1	44	0	0.714285714
1234	1	64	4	0.142857143
1235	2	20	2	0.142857143
1236	1	41	0	0.142857143
1237	1	61	3	0.428571429
1238	1	53	4	0.714285714
1239	2	60	11	0.714285714
1240	1	48	5	0.428571429
1241	1	52	6	0.142857143

1242	1	57	7	0.714285714
1243	1	73	23	0.428571429
1244	1	54	3	0.285714286
1245	1	55	3	0.142857143
1246	2	51	9	0.571428571
1247	1	55	6	0.857142857
1248	2	76	27	1
1249	2	62	6	0.428571429
1250	2	55	1	0.142857143
1251	1	57	5	0.571428571
1252	1	58	8	0.428571429
1253	2	73	10	0.714285714
1254	2	50	0	0
1255	1	27	0	0.285714286
1256	2	29	7	0.285714286
1257	1	45	12	0.428571429
1258	1	41	4	0.285714286
1259	1	96	8	0.285714286
1260	1	56	3	0.714285714
1261	2	38	5	0.428571429
1262	1	45	5	0.285714286
1263	2	63	10	0.285714286
1264	1	64	11	1
1265	1	60	5	0.428571429
1266	1	55	2	0.142857143
1267	1	62	12	1
1268	1	58	3	0.571428571
1269	1	57	6	0.428571429
1270	1	56	9	0
1271	1	66	5	0.285714286
1272	2	61	7	0.571428571
1273	1	54	3	0.571428571
1274	1	39	7	0
1275	1	59	9	0.714285714
1276	1	56	6	0
1277	1	51	0	0
1278	1	60	3	0.428571429
1279	1	57	6	0.428571429
1280	1	79	20	0.857142857
1281	1	51	3	0.285714286
1282	1	50	1	0.142857143
1283	1	60	6	0.571428571
1284	1	40	9	0.714285714
1285	1	57	5	0.285714286
1286	2	58	10	0.428571429
1287	2	50	3	0
1288	1	38	8	0.571428571
1289	1	52	6	0.714285714
1290	1	39	3	0.142857143
1291	1	57	5	0.285714286
1292	2	55	10	0.142857143
1293	1	60	6	0.285714286
1294	1	63	13	0.428571429
1295	2	34	0	0

1296	2	64	3	0.285714286
1297	1	51	6	0.285714286
1298	2	50	9	0.142857143
1299	2	60	7	0.142857143
1300	1	64	5	0
1301	2	57	5	0.571428571
1302	2	45	1	0
1303	1	53	9	0.285714286
1304	1	74	22	0.714285714
1305	1	59	9	0.571428571
1306	1	56	5	0.142857143
1307	2	40	6	0.285714286
1308	1	56	2	0.142857143
1309	1	57	9	0.142857143
1310	2	57	9	0.428571429
1311	1	51	3	0.428571429
1312	1	49	7	0.285714286
1313	1	62	6	0.142857143
1314	1	87	7	0.714285714
1315	1	51	3	0.571428571
1316	2	69	11	0.571428571
1317	1	61	5	0.285714286
1318	1	24	4	0.857142857
1319	1	56	6	0.285714286
1320	1	47	3	0.285714286
1321	1	43	9	0.285714286
1322	1	39	0	0.571428571
1323	1	61	13	0.571428571
1324	1	56	6	0.142857143
1325	2	57	7	0.428571429
1326	1	36	5	0.285714286
1327	2	56	2	0.142857143
1328	1	56	6	0.142857143
1329	2	57	9	0.428571429
1330	1	59	3	0.571428571
1331	1	56	4	0.571428571
1332	1	61	27	0.571428571
1333	2	53	9	0
1334	1	42	1	0
1335	1	55	9	0.142857143
1336	1	25	9	0.428571429
1337	1	60	11	0.142857143
1338	1	20	0	0.571428571
1339	1	49	3	0.142857143
1340	1	56	3	0.857142857
1341	1	60	9	0
1342	2	58	3	0.714285714
1343	1	52	4	0.142857143
1344	2	60	14	0.285714286
1345	2	58	6	0
1346	2	60	11	0.285714286
1347	1	59	8	0.857142857
1348	2	45	5	0.714285714
1349	1	49	0	0

1350	1	20	0	0.428571429
1351	2	55	2	0
1352	2	63	10	0.285714286
1353	1	62	6	0.142857143
1354	1	50	6	0.142857143
1355	2	55	7	0.857142857
1356	1	56	0	0.285714286
1357	1	45	0	0.428571429
1358	2	51	2	0
1359	1	47	0	0.285714286
1360	2	49	0	0.857142857
1361	2	58	7	0.285714286
1362	1	49	3	0.142857143
1363	2	58	2	0.285714286
1364	1	70	5	0.142857143
1365	1	72	11	0.571428571
1366	1	59	4	0.428571429
1367	1	57	1	0.571428571
1368	1	60	8	0
1369	2	58	7	0.571428571
1370	1	61	4	0.428571429
1371	1	60	3	0.285714286
1372	2	55	4	0
1373	1	51	0	0.571428571
1374	2	55	5	0
1375	2	59	3	0
1376	2	46	1	0.285714286
1377	2	21	9	0.285714286
1378	2	70	16	0.142857143
1379	2	59	1	0
1380	2	55	7	0.142857143
1381	1	49	0	0.285714286
1382	2	56	5	0.714285714
1383	1	52	1	0.571428571
1384	1	50	1	0.142857143
1385	1	58	16	0.714285714
1386	2	47	4	0
1387	2	60	9	0.857142857
1388	1	42	0	0.142857143
1389	2	61	6	0
1390	2	53	6	0.571428571
1391	1	41	0	0.285714286
1392	2	51	5	0.428571429
1393	1	41	0	0.857142857
1394	2	52	4	0.142857143
1395	2	42	5	0
1396	1	59	0	0
1397	1	63	13	0
1398	1	57	11	0.285714286
1399	1	56	5	0
1400	1	59	10	0
1401	2	56	0	0
1402	1	41	2	0.285714286
1403	1	57	11	0.428571429

1404	1	69	0	0.285714286
1405	1	31	0	0.571428571
1406	1	69	11	0.285714286
1407	1	72	12	0.285714286
1408	1	41	3	0.142857143
1409	2	59	6	0.428571429
1410	1	57	1	0.285714286
1411	2	57	6	0.285714286
1412	1	38	0	0.428571429
1413	1	61	12	0.285714286
1414	1	49	7	0.285714286
1415	1	50	0	0.571428571
1416	1	56	7	0.285714286
1417	1	48	4	0.142857143
1418	2	50	5	0.428571429
1419	1	41	3	0.142857143
1420	1	60	8	0.857142857
1421	1	71	12	0.142857143
1422	1	53	3	0.142857143
1423	1	63	6	0.428571429
1424	1	59	3	0.142857143
1425	1	53	6	0.285714286
1426	1	77	4	0.285714286
1427	1	27	3	0.285714286
1428	1	50	6	0.571428571
1429	2	62	14	0.285714286
1430	1	45	2	0.285714286
1431	2	56	0	0.142857143
1432	2	43	0	0.428571429
1433	1	59	5	0.285714286
1434	1	60	0	0.428571429
1435	1	55	7	0.571428571
1436	1	40	0	0.285714286
1437	1	52	9	0.428571429
1438	1	21	0	0.571428571
1439	1	60	9	0.428571429
1440	1	60	7	0
1441	1	48	4	0.428571429
1442	2	52	5	0.571428571
1443	1	48	0	0.285714286
1444	2	44	2	0.571428571
1445	1	33	0	0
1446	2	56	7	0.285714286
1447	1	58	10	0.142857143
1448	2	61	4	0.285714286
1449	1	60	2	0.285714286
1450	1	59	4	0.714285714
1451	1	62	13	0
1452	2	50	0	0.428571429
1453	2	60	1	0.285714286
1454	1	50	6	0.571428571
1455	1	59	3	0.714285714
1456	2	52	0	0.142857143
1457	1	54	6	0

1458	1	42	2	0.285714286
1459	1	31	16	0.285714286
1460	1	63	5	0.285714286
1461	2	59	9	0.142857143
1462	2	24	0	0.571428571
1463	1	47	1	0
1464	1	54	3	0.142857143
1465	1	56	6	0
1466	1	51	7	0.571428571
1467	1	58	5	0.285714286
1468	2	57	5	0.142857143
1469	1	40	5	0.142857143
1470	1	60	8	0.714285714
1471	1	59	4	0.428571429
1472	1	54	3	0.142857143
1473	1	50	0	0
1474	1	49	4	0.857142857
1475	1	30	1	0
1476	1	62	7	0.428571429
1477	1	59	11	0.142857143
1478	1	57	10	0.571428571
1479	1	53	3	0
1480	1	63	17	0.285714286
1481	1	54	1	0
1482	1	48	1	0
1483	1	48	0	0.142857143
1484	1	59	3	0
1485	2	57	8	0.571428571
1486	1	59	0	0.428571429
1487	1	50	0	0.142857143
1488	1	57	0	0.571428571
1489	1	48	5	0.428571429
1490	1	54	0	0.428571429
1491	1	56	8	0.285714286
1492	1	33	2	0.285714286
1493	1	71	15	0.714285714
1494	1	20	0	0.714285714
1495	1	43	0	0.142857143
1496	1	59	3	0.285714286
1497	2	53	2	0.857142857
1498	1	61	9	0
1499	2	46	2	0.142857143
1500	1	52	14	0.571428571
1501	1	53	4	0
1502	1	58	4	0
1503	1	34	1	0.142857143
1504	1	54	0	0.285714286
1505	1	61	5	0.571428571
1506	1	30	1	0.857142857
1507	1	53	2	0.285714286
1508	1	54	3	0.142857143
1509	1	58	4	0
1510	1	44	2	0.285714286
1511	1	55	0	0.857142857

1512	1	40	1	0
1513	1	45	6	0.571428571
1514	1	20	0	0
1515	1	60	5	0.285714286
1516	1	21	0	0.142857143
1517	2	69	2	0.285714286
1518	1	63	9	0.285714286
1519	1	46	0	0
1520	1	49	5	0.428571429
1521	1	56	6	0.142857143
1522	1	58	9	0.285714286
1523	2	67	6	0.428571429
1524	1	20	1	0
1525	2	38	2	0.571428571
1526	1	61	13	0.428571429
1527	1	60	21	0.571428571
1528	1	60	6	0.142857143
1529	1	61	7	0.142857143
1530	1	52	5	0.285714286
1531	1	60	7	0.285714286
1532	1	49	0	0
1533	1	44	1	0
1534	1	65	13	0
1535	1	54	1	0.142857143
1536	1	47	2	0.142857143
1537	1	76	18	0.285714286
1538	1	39	1	0.428571429
1539	2	61	0	0.428571429
1540	1	34	3	0.285714286
1541	1	46	9	0.571428571
1542	1	56	0	0.285714286
1543	1	61	3	0.142857143
1544	1	46	0	0.714285714
1545	1	64	7	0.428571429
1546	1	54	2	0.142857143
1547	1	62	4	0
1548	1	46	4	0.571428571
1549	1	33	3	0.714285714
1550	2	74	19	0.571428571
1551	1	60	7	0.285714286
1552	1	45	3	0.142857143
1553	2	44	0	0
1554	1	46	0	0
1555	1	56	7	0.285714286
1556	1	53	7	0.428571429
1557	2	54	0	0.142857143
1558	1	60	7	0.142857143
1559	2	34	0	0.571428571
1560	2	70	10	0.714285714
1561	1	50	3	0.142857143
1562	1	56	7	0.142857143
1563	2	42	0	0
1564	1	41	0	0.428571429
1565	1	68	5	0

1566	1	57	9	0.285714286
1567	2	47	0	0
1568	1	59	4	0
1569	1	43	11	0.285714286
1570	2	69	19	0.285714286
1571	2	39	0	0.571428571
1572	2	73	10	0.857142857
1573	1	62	18	0.142857143
1574	1	60	7	0.428571429
1575	1	56	1	0
1576	1	26	0	0.285714286
1577	1	60	12	0.428571429
1578	1	53	4	0.571428571
1579	1	44	0	0.428571429
1580	1	47	13	0.428571429
1581	1	63	8	0.571428571
1582	1	62	17	0.285714286
1583	1	46	5	0.571428571
1584	2	61	12	0.142857143
1585	2	35	9	0
1586	2	45	0	0
1587	1	67	4	0.428571429
1588	2	40	2	0.285714286
1589	1	53	1	0.428571429
1590	1	62	8	0.285714286
1591	1	46	1	0
1592	2	65	6	0.428571429
1593	1	48	2	0.571428571
1594	2	45	0	0.428571429
1595	1	48	7	0.285714286
1596	1	48	9	0.285714286
1597	1	58	10	0.428571429
1598	2	58	6	0.428571429
1599	1	67	17	0.285714286
1600	2	40	1	0
1601	1	44	4	0.142857143
1602	2	64	0	0.428571429
1603	1	49	7	0.142857143
1604	1	53	0	0
1605	1	43	0	0.428571429
1606	2	46	3	0.285714286
1607	2	58	10	0
1608	1	37	6	0.142857143
1609	2	56	2	0.428571429
1610	2	59	9	0.571428571
1611	1	45	3	0.428571429
1612	2	60	3	0
1613	2	49	6	0.285714286
1614	2	59	11	0.142857143
1615	1	20	1	0.571428571
1616	2	60	0	0.142857143
1617	1	53	0	0.142857143
1618	2	52	5	0.142857143
1619	1	47	0	0.142857143

1620	2	66	6	0.142857143
1621	1	20	0	0.285714286
1622	1	61	7	0.285714286
1623	2	65	2	0.142857143
1624	1	52	7	0.857142857
1625	2	51	7	0.714285714
1626	1	60	9	0.285714286
1627	2	64	2	0.571428571
1628	2	35	0	0.142857143
1629	1	59	10	0.571428571
1630	1	43	0	0.428571429
1631	1	60	6	0.571428571
1632	2	57	0	0.285714286
1633	1	58	5	0.285714286
1634	1	43	0	0
1635	1	65	12	0.285714286
1636	2	49	2	0.857142857
1637	1	48	7	0.285714286
1638	2	59	8	0.285714286
1639	1	42	0	0.142857143
1640	1	40	3	0.142857143
1641	2	58	5	0.285714286
1642	1	47	5	0.857142857
1643	2	57	8	0.428571429
1644	2	58	0	0.428571429
1645	2	60	4	0
1646	2	57	5	0.285714286
1647	1	67	12	0.142857143
1648	1	69	13	0.142857143
1649	2	55	0	0.285714286
1650	2	34	0	0.857142857
1651	1	46	4	0.142857143
1652	2	53	2	0.285714286
1653	2	50	1	0.571428571
1654	2	48	5	0.285714286
1655	1	54	5	0
1656	2	46	5	0.142857143
1657	2	57	3	0
1658	1	48	6	0
1659	2	61	24	0.142857143
1660	2	48	0	0
1661	1	41	0	0.428571429
1662	1	39	0	0.714285714
1663	2	56	5	0.142857143
1664	2	43	0	0
1665	1	43	2	0.428571429
1666	2	42	4	0.142857143
1667	2	57	10	0.428571429
1668	1	52	3	0.285714286
1669	2	37	0	0
1670	2	62	10	0
1671	2	51	5	0.285714286
1672	2	46	3	0
1673	2	51	1	0

1674	1	44	6	0.142857143
1675	2	55	4	0.142857143
1676	2	44	4	0.142857143
1677	1	52	6	0.714285714
1678	2	53	6	0.857142857
1679	1	53	2	0.428571429
1680	2	63	4	0.142857143
1681	2	55	4	0.428571429
1682	2	46	0	0.142857143
1683	1	36	1	0.142857143
1684	1	45	2	0.142857143
1685	2	53	10	0.285714286
1686	2	49	3	0.857142857
1687	1	67	13	0.285714286
1688	2	39	2	0.428571429
1689	2	56	3	0.285714286
1690	1	55	4	0.142857143
1691	2	48	5	0.285714286
1692	2	63	8	0
1693	1	57	5	0.428571429
1694	2	55	3	0
1695	2	46	0	0.285714286
1696	2	58	3	0
1697	2	50	4	0
1698	2	56	8	0.142857143
1699	2	51	0	0.428571429
1700	2	58	8	0.142857143
1701	2	56	1	0.285714286
1702	2	54	3	0.714285714
1703	2	62	4	0.285714286
1704	1	56	2	0.142857143
1705	2	58	8	0
1706	2	64	3	0.428571429
1707	2	48	7	0
1708	1	59	7	0.285714286
1709	2	58	9	0.857142857
1710	2	50	1	0.714285714
1711	2	53	3	0.285714286
1712	2	57	10	0
1713	2	54	4	0.714285714
1714	1	60	7	0.285714286
1715	2	62	3	0.285714286
1716	1	47	14	0.428571429
1717	2	43	3	0
1718	2	58	21	0.571428571
1719	2	44	6	0.571428571
1720	2	50	7	0.142857143
1721	2	52	7	0.285714286
1722	2	28	2	0.142857143
1723	2	49	2	0.142857143
1724	2	42	1	0.428571429
1725	2	51	4	0.285714286
1726	2	68	5	0.142857143
1727	1	38	3	0.714285714

1728	2	70	9	0
1729	2	56	3	0.714285714
1730	2	50	0	0.142857143
1731	2	58	3	0.571428571
1732	1	53	9	0.142857143
1733	2	61	0	0.285714286
1734	2	55	1	0
1735	2	45	6	0.714285714
1736	2	66	5	0
1737	2	45	3	0.285714286
1738	2	57	16	0.285714286
1739	2	67	8	0.428571429
1740	2	57	8	0.857142857
1741	2	57	5	0.857142857
1742	2	54	1	0.857142857
1743	2	44	0	0.857142857
1744	2	60	1	0.142857143
1745	1	47	1	0
1746	2	59	3	0
1747	1	59	8	0.285714286
1748	1	42	3	0.142857143
1749	2	65	7	0.571428571
1750	2	40	0	0.285714286
1751	2	56	9	0.714285714
1752	2	61	8	0.285714286
1753	2	41	1	0.714285714
1754	2	41	3	0.571428571
1755	1	50	0	0.285714286
1756	2	55	0	0.428571429
1757	2	64	6	0.428571429
1758	2	57	7	0.428571429
1759	2	51	2	0.142857143
1760	2	47	0	0.142857143
1761	1	61	4	0.428571429
1762	1	47	0	0.428571429
1763	1	55	4	0.142857143
1764	2	60	4	0.142857143
1765	2	56	11	0.428571429
1766	2	70	16	0
1767	1	57	5	0.714285714
1768	2	47	0	0.571428571
1769	2	54	4	0.142857143
1770	2	57	2	0.571428571
1771	1	49	3	0
1772	1	42	1	0.285714286
1773	2	54	4	0
1774	1	43	2	0.428571429
1775	2	87	18	0.142857143

group（1=l编号		age	PHQ-9	U	D	moral acceptance
DE	1	19	26	0	1	1.428571429
DE	2	19	21	0.5714286	1	3.285714286
DE	3	20	22	0.2857143	0.4	4
DE	4	19	20	0.7142857	0.5	4
DE	5	21	20	0	0.7142857	4
DE	6	18	20	0.2857143	1	3.571428571
DE	7	19	27	0.2857143	0.8	3.571428571
DE	8	18	22	-0.428571	0.7	2
DE	9	19	20	0	0.8571429	1.857142857
DE	10	19	24	0.2857143	0.6	4.714285714
DE	11	20	26	0	0.4285714	3.571428571
DE	12	19	24	0.1428571	0	4.428571429
DE	13	19	21	0.4285714	0.5	5.285714286
DE	14	19	23	0.1428571	0.8333333	3.571428571
DE	15	21	27	-0.285714	0.2222222	6.142857143
DE	16	19	22	0	1	3.714285714
DE	17	17	24	-0.142857	0.875	3.142857143
DE	18	18	22	0	1	4
DE	19	20	25	0.1428571	0.3333333	5.571428571
DE	20	20	20	0	0.8571429	2.428571429
DE	21	18	22	0.5714286	1	2.714285714
DE	22	19	27	-0.142857	0.25	1.571428571
DE	23	19	23	0	1	1.428571429
DE	24	20	25	0.4285714	0.75	2.714285714
DE	25	18	23	0.2857143	0.8	4
DE	26	19	21	0	1	4
DE	27	20	21	0	1	4
DE	28	19	25	0.5714286	0.3333333	5.285714286
DE	29	19	21	0.1428571	0.8333333	2.285714286
DE	30	19	20	0.1428571	0.5	3.857142857
DE	31	19	23	0.2857143	1	3.571428571
DE	32	19	20	0.1428571	0.6666667	2.857142857
DE	33	19	21	0.1428571	0.5	4
DE	34	21	27	-0.285714	0.2222222	6.142857143
DE	35	20	25	0.2857143	0.6	3
HC	36	19	2	0	1	4
HC	37	21	2	0.1428571	1	1.428571429
HC	38	20	3	0	1	3
HC	39	18	2	0	1	2.142857143
HC	40	20	0	0	0.1428571	1.142857143
HC	41	19	0	0	1	1.857142857
HC	42	18	0	0.2857143	1	1.142857143
HC	43	19	3	0.1428571	0.8333333	3.142857143
HC	44	20	0	0.1428571	1	2
HC	45	20	0	0	0.7142857	2.428571429
HC	46	18	0	0.1428571	0.8333333	2.428571429
HC	47	19	0	0.1428571	0.5	2.285714286
HC	48	19	1	0.4285714	1	4
HC	49	20	2	0.1428571	1	2.142857143
HC	50	20	2	-0.142857	0.875	1
HC	51	19	0	0	0.8571429	2.285714286
HC	52	19	3	0.2857143	0.8	4.428571429
HC	53	19	3	0.4285714	0.75	2.714285714

HC	54	19	2	0.2857143	1	2.714285714
HC	55	19	3	0.4285714	0.75	3.428571429
HC	56	19	1	0.1428571	1	1.714285714
HC	57	21	1	0	0.8571429	3.285714286
HC	58	19	2	0	0.7142857	2.714285714
HC	59	19	0	0	1	1.285714286
HC	60	20	0	0.2857143	1	3.142857143
HC	61	19	0	0.2857143	0	1.857142857
HC	62	19	2	0.2857143	1	3.142857143
HC	63	19	3	0	0.8571429	2.285714286
HC	64	20	2	-0.142857	0.875	1
HC	65	20	3	0.2857143	1	2.571428571
HC	66	18	2	0	1	1.428571429
HC	67	20	3	0	0.8571429	3.285714286
HC	68	18	1	0.1428571	1	1.714285714
HC	69	20	5	0.2857143	0.6	4
HC	70	20	2	0	0.5714286	3.571428571
